# Body Composition of Male Professional Soccer Players Using Different Measurement Methods: A Systematic Review and Meta-Analysis

**DOI:** 10.3390/nu15051160

**Published:** 2023-02-25

**Authors:** Jaime Sebastiá-Rico, Jose M. Soriano, Noelia González-Gálvez, José Miguel Martínez-Sanz

**Affiliations:** 1 Area of Nutrition, University Clinic of Nutrition, Physical Activity and Physiotherapy (CUNAFF), Lluís Alcanyís Foundation-University of Valencia, 46020 Valencia, Spain; 2 Food and Nutrition Research Group (ALINUT), University of Alicante, 03690 Alicante, Spain; 3 Food & Health Lab, Institute of Materials Science, University of Valencia, 46980 Paterna, Spain; 4 Joint Research Unit of Endocrinology, Nutrition and Clinical Dietetics, University of Valencia-Health Research Institute La Fe, 46026 Valencia, Spain; 5 Sports Injury Prevention Research Group, Faculty of Sport, Catholic University of Murcia (UCAM), 30107 Murcia, Spain; 6 Nursing Department, Faculty of Health Sciences, University of Alicante, 03690 Alicante, Spain

**Keywords:** soccer, body composition, anthropometry, bioimpedance, DXA

## Abstract

The performance of male soccer players (MSP) depends on multiple factors such as body composition. The physical demands of modern soccer have changed, so the ideal body composition (BC) requirements must be adapted to the present. The aim of this systematic review and meta-analysis was to describe the anthropometric, BC, and somatotype characteristics of professional MSP and to compare the values reported according to the methods and equations used. We systematically searched Embase, PubMed, SPORTDiscus, and Web of Science following the PRISMA statement. Random-effects meta-analysis, a pooled summary of means, and 95% CI (method or equation) were calculated. Random models were used with the restricted maximum likelihood (REML) method. Seventy-four articles were included in the systematic review and seventy-three in the meta-analysis. After comparing the groups according to the assessment method (kinanthropometry, bioimpedance, and densitometry), significant differences were found in height, fat mass in kilograms, fat mass percentage, and fat-free mass in kilograms (*p* = 0.001; *p* < 0.0001). Taking into account the equation used to calculate the fat mass percentage and ∑skinfolds, significant differences were observed in the data reported according to groups (*p* < 0.001). Despite the limitations, this study provides useful information that could help medical technical staff to properly assess the BC of professional MSP, providing a range of guidance values for the different BC.

## 1. Introduction

Kinanthropometry is the area of science responsible for measuring the composition of the human body. Changes in lifestyle, nutrition, physical activity, and ethnic composition of populations are some of the factors that can cause alterations in body dimensions [1]. In sports (including soccer), anthropometry, bioelectrical impedance analysis (BIA) and dual X-ray absorptiometry (DXA) are the main methods used to assess body composition [2,3].

Anthropometry refers to the different measurements taken of the size and proportions of the human body by which, through equations, an estimation of the percentage of fat mass (FM), and by derivation, the fat-free mass (FFM), can be obtained [4]. This evaluation method has been described by different institutions, with the International Society for the Advancement of Kinanthropometry (ISAK) being the gold standard institution, according to ISO 7250-1:2017 and the ISAK standard [1]. This method is useful for nutritional and training control and monitoring [2,3].

BIA is a non-invasive and easy-to-apply method based on the principle that states that the conductivity of body water varies between the different compartments and can therefore be used for the calculation of total body water, FM, and FFM [5,6]. The method consists of measuring the resistance of the body to the flow of a current. In the adipose compartments, a greater resistance will be observed as these compartments are poorer conductors of electricity due to their low water volume, whereas muscle tissues, as they have a high-water content, together with a high concentration of electrolytes, will act as good electrical conductors [4].

DXA is an indirect method used to measure muscle mass (MM), FM, and bone mineral density (BMD) through photon attenuation (X-rays). Soft tissues, due to their high water and organic compound content, reduce the photon flux to a lesser extent than bone tissue, and the pixels of the bone compartment are more clearly distinguishable [6]. It is therefore considered the gold standard method for the assessment of bone mineral status [7,8].

Another of the most relevant tools used in the study of body composition is somatotype, defined as the quantification of the shape and composition of the human body through the numerical quantification of three components, by using different anthropometric formulas and measurements [9]. Derived from the somatotype, the somatochart is the expression of the three components (endomorphy, mesomorphy, and ectomorphy) as a graphic representation. In the field of sports, it is useful to be able to compare the somatotype of the athlete being evaluated against the reference somatotype of the sport he/she practices, based on a wide collection of data [10].

Therefore, the assessment of body composition plays a crucial role in athletes, as it directly affects both performance and sporting results in competitions [11]. In soccer, body composition is crucial for achieving an optimal physical level, which can translate into a good level of play, as performance in soccer depends on multiple technical, biomechanical, tactical, mental, and physiological factors, as well as nutritional and training control [12].

The aim of the present systematic review with meta-analysis was to describe the anthropometric characteristics, body composition, and somatotype of professional male soccer players, and to compare the values reported according to the methods and equations used. Consequently, the initial hypotheses were:

**Hypothesis 1 (H1).**  *Depending on the measurement instrument used, different values of body composition will be observed for the same compartment, especially in the weight and percentage of the FM.*

**Hypothesis 2 (H2).**  *There will be differences between anthropometric equations for the same body compartment.*

## 2. Materials and Methods

### 2.1. Type of Study

This systematic review and meta-analysis is based on existing evidence on anthropometric characteristics, body composition, and somatotype, of professional male soccer players. It was conducted based on the Preferred Reporting Items for Systematic Reviews and Meta-Analyses (PRISMA) [13].

### 2.2. Information Sources and Search Strategy

The databases searched to obtain the most current data were PubMed-MEDLINE, Embase, SPORTDiscus, and Web of Science. To find the largest number of available articles related to the research aim, the words used in the search strategy were defined considering: (1) soccer (football); (2) anthropometry, body composition and somatotype; (3) athlete (professional or elite); (4) the descriptors of the Medical Subjects Headings (MeSH); (5) other terms described in MeSH as “entry terms”, which include the terminology prior to the setting-up of the MeSH register; and (6) the terms (tiab) or (Title/Abstract) attached to the “entry terms” or MeSH, which allow the localization of these terms in the title and abstract of the articles. The search strategy used for PubMed was: (“Soccer” (Mesh) OR “soccer player” (Title/Abstract) OR “fútbol” (Title/Abstract) OR “soccer” (Title/Abstract) OR “football” (Title/Abstract)) AND (“Anthropometry” (MeSH) OR “Anthropometry” (Title/Abstract) OR “Body composition” (MeSH) OR “Body composition” (Title/Abstract) OR “Skinfolds” (Title/Abstract) OR “Skinfold Thickness” (MeSH) OR “Somatotypes” (Mesh) OR “Somatotypes” (Title/Abstract) OR “Body Build” (Title/Abstract) OR “Body Type” (Title/Abstract) OR “Endomorph” (Title/Abstract) OR “Mesomorph” (Title/Abstract) OR “Ectomorph” (Title/Abstract) OR “Absorptiometry, Photon” (Mesh) OR “Absorptiometry, Photon” (Title/Abstract) OR “Electric Impedance” (Mesh) OR “Electric Impedance” (Title/Abstract) OR “Bioimpedance” (Title/Abstract) OR “DXA” (Title/Abstract) OR “Dual Energy X-ray Absorptiometry” (Title/Abstract)) NOT (“youth” (Title/Abstract) OR “young” (Title/Abstract) OR “semi-professional” (Title/Abstract) OR “amateur” (Title/Abstract) OR “collegiate” (Title/Abstract) OR “pre-adolescent” (Title/Abstract) OR “recreational” (Title/Abstract) OR “adolescent” (Title/Abstract) OR “junior” (Title/Abstract) OR “referee” (Title/Abstract) OR “referees” (Title/Abstract) OR “gaelic” (Title/Abstract) OR “rugby” (Title/Abstract) OR “american football” (Title/Abstract) OR “female” (Title/Abstract) OR “women” (Title/Abstract)) AND (“professional” (Title/Abstract) OR “elite” (Title/Abstract)).

The search strategy was adapted for each of the databases consulted through the Polyglot Search of the Systematic Review Accelerator tool (accessed on 1 December 2021. https://sr-accelerator.com/#/polyglot).

The timeframe for the search included studies from the year 2000 until November 2021. Due to the fact that the physical demands of soccer players have evolved over the decades, they are currently more demanding and require different body composition characteristics [14].

### 2.3. Eligibility Criteria

The Participants, Intervention, Comparison, and Outcomes (PICO) criteria for the inclusion and exclusion criteria are shown on Table 1. No limits were placed in relation to the publication status of the study (pre-print, post-print, first online, or final).

The exclusion criteria included: (a) studies published in a language other than Spanish and/or English, and (b) narrative, systematic reviews, and/or meta-analyses.

### 2.4. Article Management Process

All the documents found were incorporated into the Zotero citation manager in a separate folder, depending on the database where they were found. A common folder was created to detect and delete duplicated articles using the software’s degree of data overlap. The final database was exported in RIS format to be imported into the article screening system for further processing by the researchers.

### 2.5. Study Selection

All retrieved articles were screened in duplicate. The first screening, based on the title and abstract, was independently conducted in all the studies by two authors (JS-R and JMM-S). During the processes of identifying and screening, a third researcher was consulted (JMS) to determine if the documents that led to discrepancies between authors had to be included or excluded. The articles eligible for a full-text review were then screened by the same authors (JS-R and JMM-S), independently and in duplicate. The rejected articles were then duly identified using the eligibility criterion previously established. Additional reviewers (JMS, NG-G) provided advice when feedback about doubtful documents was required.

### 2.6. Data Extraction

The studies’ information was extracted following a blinded and duplicated protocol by two authors (JS-R and JMM-S) using a previously piloted data extraction survey created for this review. The data extraction protocol for this study consisted of the following variables:

- Study: Authors and year of publication
- Country and competition category: Geographical area and competitive category from which the data comes. The latter was included to differentiate between professional league categories within the same country.
- Sample: Number of subjects.
- Time of Season: Included to differentiate values collected between different cycles of a natural season (if specified).
- Method of analysis: It was included to differentiate the values collected between the three methods of evaluation of body composition.
- Measuring instruments: description of the material used in the evaluation.
- Main results: Kinanthropometric characteristics and values of FM, MM, bone mass (BM), and body water

### 2.7. Study Quality and Data Collection

Two researchers (JS-R and JMM-S) examined the quality of the studies using the Agency for Health Research and Quality (AHRQ) Methodology Checklist [15]. A third reviewer (JMS) was consulted to resolve discrepancies. A score above 8 was considered a high-quality study. Egger’s bias statistic [16] was used to assess the risk of bias, and funnel plots were created. When a meta-analysis was based on a small number of studies, the ability of Egger’s test to detect bias is limited [17]. Therefore, this test was only performed when there were at least 10 studies included in the meta-analysis [16]

### 2.8. Statistical Analysis

The meta-analysis was performed with the R software version 3.6.0. Copyright (C) 2019 (R Foundation for Statistical Computing). The meta-analysis was performed for continuous data using sample (n), mean (M), and standard deviation (SD) of each output from each study. Some studies had more than one group and were treated as other subgroups in the analysis. In the random-effects meta-analysis, a pooled summary mean, and 95%CI were calculated. Studies were weighted according to sample within and between studies. A pooled summary mean and 95%CI were calculated for subgroups (method or equation) in order to compare the differences between groups. Random models using the restricted maximum likelihood method (REML) were utilized. The heterogeneity was measured using the I^2^ statistic, considering a high heterogeneity if I^2^ ≥ 75%. The level of significance adopted was 5% (*p* < 0.05).

## 3. Results

**Figure 1:**
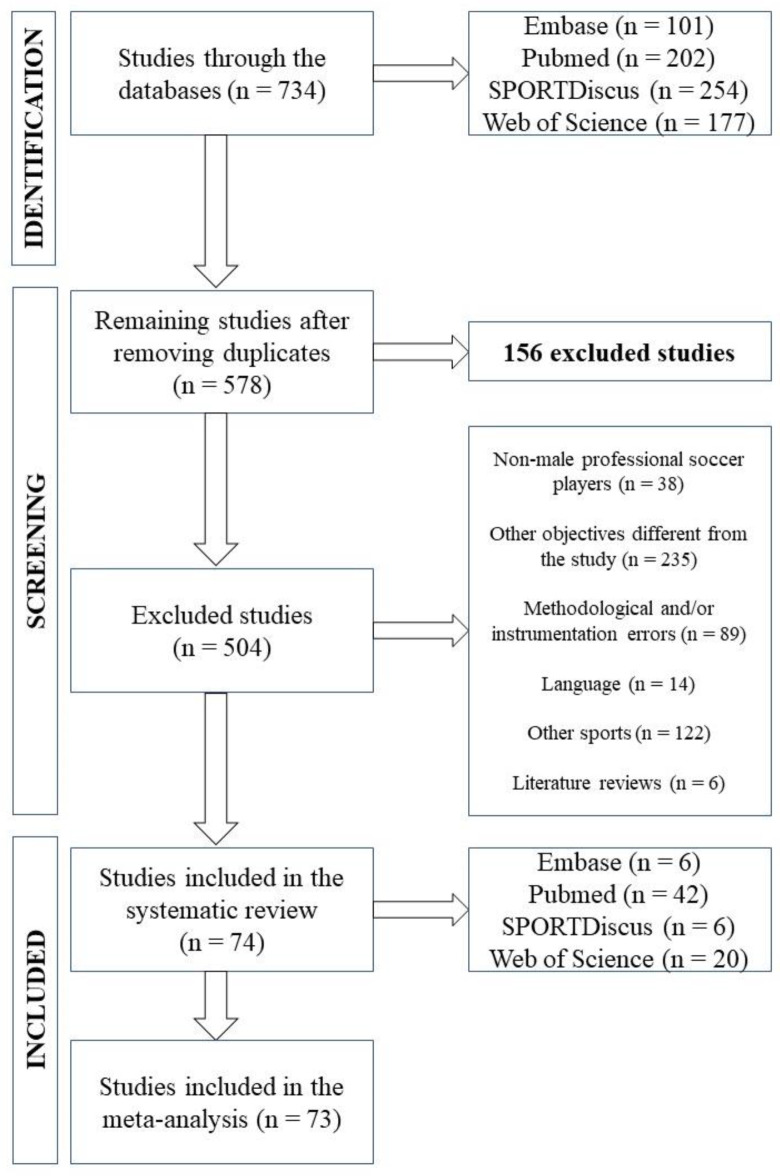
Flow diagram showing the process used to select the studies.

A total of 74 studies were included in the systematic review, of which 32 were based on the use of anthropometry [18,19,20,21,22,23,24,25,26,27,28,29,30,31,32,33,34,35,36,37,38,39,40,41,42,43,44,45,46,47,48,49], 21 in BIA [50,51,52,53,54,55,56,57,58,59,60,61,62,63,64,65,66,67,68,69,70], 13 in DXA [71,72,73,74,75,76,77,78,79,80,81,82,83], 3 combined anthropometry and BIA [84,85,86], 3 combined anthropometry and DXA [87,88,89], and 2 combined BIA and DXA [90,91], while 73 articles were included in the meta-analysis (Figure 1).

Table 2, Table 3 and Table 4 show the qualitative characteristics of the included articles differentiated by measurement methods (anthropometry, BIA, and DXA, respectively). Table 5 shows the evaluation of methodological quality as assessed with the Methodology Checklist from the Agency for Health Research and Quality (AHRQ) [15].

The sample comprising the different articles included in the review amounted to a total of 5197 soccer players.

In relation to the anthropometric measuring instrument, the main one used was the Harpenden plicometer (*n* = 25) [18,19,20,21,23,24,25,26,28,31,33,35,36,37,39,40,41,44,46,47,48,86,87,88,89], followed by the Holtain (*n* = 8) [22,29,34,38,42,43,84,85] and the Slimguide (*n* = 5) [27,30,32,45,49] ones. For BIA, the most commonly used models were the Tanita BC 418 MA (*n* = 5) [58,62,64,67,86], Akern BIA 101 (*n* = 4) [57,65,84,85], and Tanita TBF 543 (*n* = 2) [52,60]. Lastly, the most common DXA machine models were GE Lunar Prodigy (*n* = 4) [73,87,89,90], Hologic QDR Series Delphi A (*n* = 2) [82,91], Hologic QDR Series Discovery A (*n* = 2) [71,76] and Hologic Discovery W (*n* = 2) [78,79].

Of the 38 articles included in which anthropometry was used as a method of assessment, 15 used the ISAK protocol [22,28,30,31,32,34,35,41,43,45,49,85,86,88,89], 2 utilized the protocol from the Anthropometric Standardization Reference Manual (ASRM) [29,38], 1 used the protocol from the International Biological Program (IBP) [47], 1 used the protocol from the American College of Sports Medicine (ACSM) [46], and the remaining 19 articles did not specify the protocol applied [18,19,20,21,23,24,25,26,27,33,36,37,39,40,42,44,48,84,87].

As for the sum of skinfolds, the most commonly used formulas were the sum of six skinfolds (triceps, subscapular, supraspinale, abdominal, mid-thigh, and calf) (*n* = 4) [22,32,34,45], followed by the sum of eight folds (biceps, triceps, subscapular, iliac crest, supraspinale, abdominal, mid-thigh, and calf) (*n* = 3) [31,40,41].

Regarding the percentage of FM, the most commonly used formula was that of Durnin and Womersley, 1974 (*n* = 10) [19,21,23,24,25,26,31,48,87,89], followed by Jackson and Pollock’s seven folds, 1978 (*n* = 6) [36,39,46,47,87,89], and Faulkner, 1968 (*n* = 4) [27,34,37,89]. In the case of the MM percentage, the most commonly used formula was Matiegka, 1921 (*n* = 3) [28,34,44], followed by Kerr, 1988 (*n* = 1). Finally, for the percentage of bone mass (BM), the two equally used formulas were those of Matiegka, 1921 and Kerr, 1988 (*n* = 1) [44,89]. Tables S1-S3 show the body composition characteristics of included studies for each of the methods analyzed.

Table 6 and Supplementary Figures S1–S9 show the meta-analysis grouped according to the method of variable assessment (1 = anthropometry, 2 = BIA, 3 = DXA) mean, standard deviation, sample size, weight, and subgroup meta-analysis (pooled summary mean, 95%CI, Tau2, Chi2, *df*, *p*, I^2^) and for the total (pooled summary mean, 95%CI, Tau2, Chi2, *df*, *p*, *I*^2^ and test for subgroup differences: Chi2, *df*, *p*). The sample had a mean age of 24.50 years old, and the following mean values were found: 179.76 cm in height, 76.29 kg in weight, 12.48 kg of FM, 11.85% of FM, 66.10 kg of FFM, 81.36% of FFM, 39.28 kg of MM and 52.03% of MM. The comparison of the groups according to the assessment method (anthropometry, BIA, and DXA) did not show significant differences in age (*p* = 0.45), (Supplementary Figure S1), weight (*p* = 0.11) (Supplementary Figure S3), percentage of FFM (*p* = 0.59) (Supplementary Figure S7), kilograms of MM (*p* = 0.37) (Supplementary Figure S8) or percentage of MM (*p* = 0.38) (Supplementary Figure S9). However, there were significant differences in height, with method 2 (BIA) showing the greatest height (*p* = 0.001) (Supplementary Figure S2); in kilograms of FM, with method 1 showing the highest value (*p* < 0.0001) (Supplementary Figure S4), in the percentage of FM, with method 3 showing the highest value (*p* < 0.0001) (Supplementary Figure S5), in kilograms of FFM, with method 1 showing the highest value (*p* = 0.0004) (Supplementary Figure S6).

**Figure 2:**
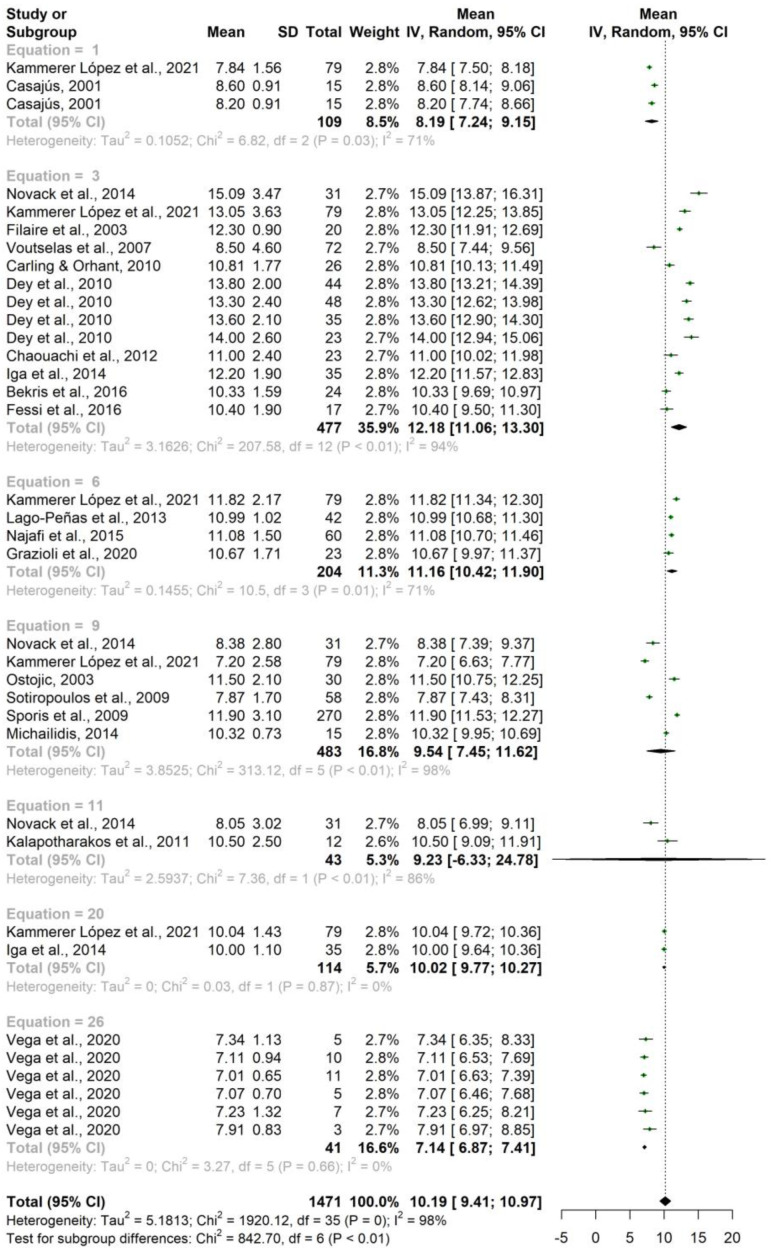
Forest plot fat mass percentage according to equation.

Taking into account the equation used for the calculation of the percentage of FM, a mean of 10.19% was obtained, with significant differences in the data reported according to groups (*p* < 0.001), with equations 3 (Durnin and Womersley, 1974) and 6 (Faulkner, 1968) indicating the highest values, and equations 1 (Carter, 1982) and 26 (Yuhasz, 1974) the lowest values (Table 7 and Figure 2).

**Figure 3:**
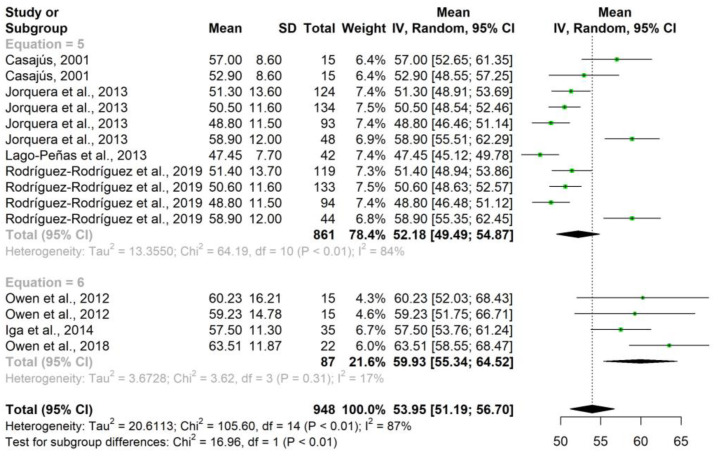
Forest plot sum of folds according to equation.

According to the equation used to calculate the sum of skinfolds, Equation (5) (triceps, subscapular, supraspinale, abdominal, anterior thigh, calf) showed a mean of 52.18 mm, while Equation (6) (triceps, biceps, subscapular, iliac crest, supraspinale, abdominal, anterior thigh, calf) showed a value of 59.93 mm (Table 7 and Figure 3).

**Figure 4:**
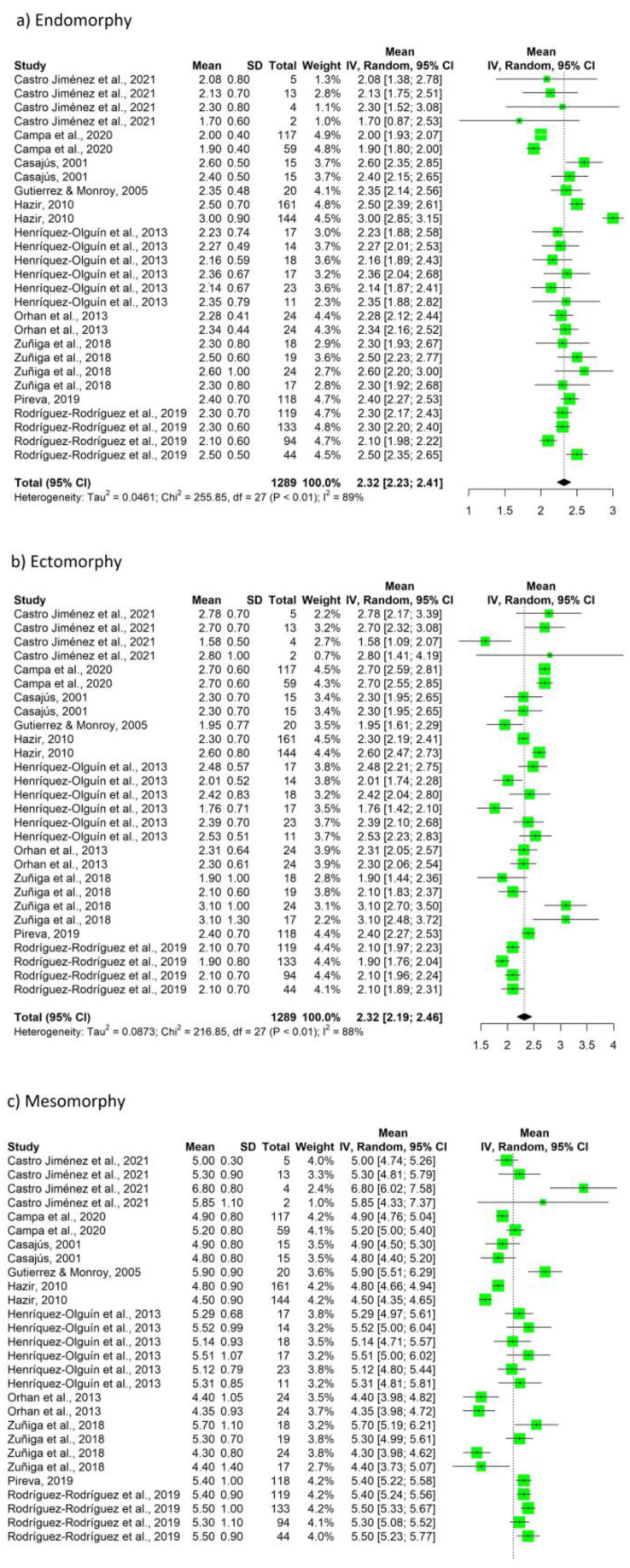
Forest plot (**a**) endomorphy, (**b**) ectomorphy, and (**c**) mesomorphy.

**Figure 5:**
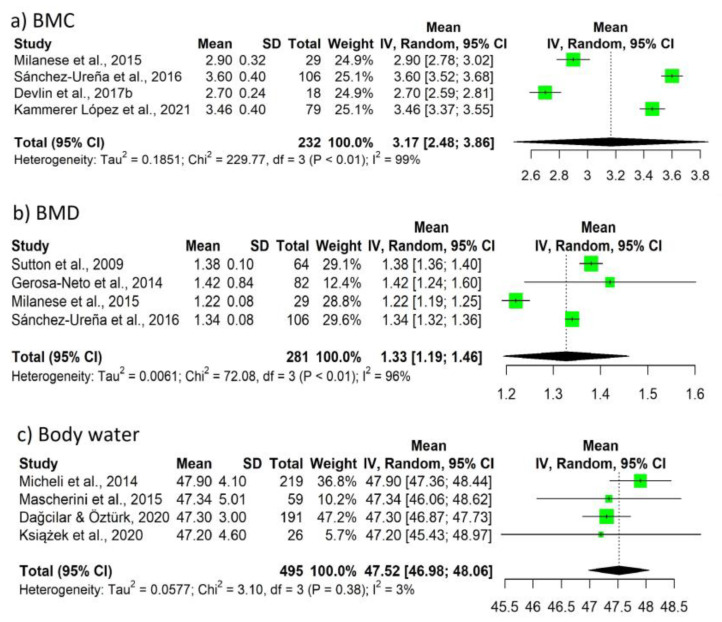
Forest plot (**a**) BMC, (**b**) BMD, and (**c**) body water.

Likewise, the data (pooled summary mean, 95%CI, Tau2, Chi2, *df*, *p*, *I*^2^) for endomorphy, ectomorphy, and mesomorphy are shown in Figure 4. The studies report a mean of 2.32 points for endomorphy, 2.32 points for ectomorphy, and 5.15 points for mesomorphy. Figure 5 shows the data for bone mineral content (BMC), BMD, and water body. With respect to BMC, mean values of 3.17 kg were reported, with respect to BMD values, 1.33 g/cm^3^, and with respect to water values, 47.52 l mean values were reported.

## 4. Discussion

This is the first systematic review with meta-analysis to assess the body composition of professional male soccer players, as well as the influences of the measurement method used. The main findings of this work were: (1) there are differences in the FM assessed between the different methods, with higher percentages using DXA followed by BIA and anthropometry, with a mean value of 11.85%; (2) there are significant differences between the different anthropometric formulas to assess the percentage of FM, with higher values in Durnin and Womersley, 1974 and Faulkner, 1968, and lower values in Yuhasz, 1974 and Carter, 1982; (3) no significant differences were observed in the measurement of MM through anthropometry and BIA, with a mean of 52.03% and 39.28 kg; (4) the weight of the FFM was higher with anthropometry, followed by BIA and DXA, with a mean value of 66.10 kg, although no significant differences were observed in the percentage of FFM through BIA and DXA, with a mean of 82.36%; (5) the mean somatotype was balanced mesomorphic.

### 4.1. Measurement Instruments

The anthropometric method is one of the most widely used methods in soccer to assess body composition, as it is inexpensive, easy to transport, non-invasive, simple to apply, and validated by the scientific community, although it requires the anthropometrist to be trained and qualified [6,8]. There are many types of plicometers available on the market, some of which are cheap and accessible, but not valid because they do not meet the technical specifications in relation to the pressure exerted on the subcutaneous tissue and/or an erroneous calibration [4,92]. In our work, the Harpenden model was the most commonly used, followed by the Holtain and Slimguide ones. This coincides with the literature, as Harpenden is the most traditionally used model in scientific research, being considered the gold standard method [93,94]. Recently, the agreement of these three analog models, together with a fourth digital model, Lipowise, was evaluated to establish the differences between the sum of skinfolds, and the estimation of FM and adipose tissue using different formulas. The authors concluded that the measurement data were similar, although the Holtain and Slimguide models were more similar to each other and tended to overestimate the result as compared to the Harpenden model [95]. It is therefore recommended that measurements be made with the same model of plicometer to monitor an individual or to compare the measured results with other studies [8,95,96].

In addition, there are many formulas for estimating the different compartments of body composition, which have an impact on the results obtained, and which are heterogeneous and not comparable with each other [97,98]. Recently, Martínez-Ferran et al. tested 21 professional soccer players from the Spanish league to discover which FM formula and sum of skinfolds correlated best with DXA as the gold standard method when assessing FM. The formula proposed by Suarez-Arrones et al., 2018, and the sum of four skinfolds (triceps, subscapular, supraspinale, and abdominal) were found to have the highest agreement [99]. In addition, this formula required only the triceps and iliac crest fold, which could reduce the time spent on the anthropometric assessment of soccer teams that are assessed on the same day and at different times in the season [99]. This correlation has been previously investigated in soccer players [89,91,100], but their results pointed to a higher correlation to formulas such as Faulkner, 1968 [101], Eston, 2005 [102], Withers et al., 1987 [103] or Durnin and Womersley, 1974 [104]. In our work, neither of the two proposals by Martínez-Ferran et al. were included, with the formula from Durnin and Womersley, 1974 [104], and the sum of six skinfolds (triceps, subscapular, supraspinale, abdominal, mid-thigh, and calf) being the most widely used in the studies included. For this reason, caution should be exercised in the selection of the formulas to be used when assessing soccer players, and the validity of the proposals by Martínez-Ferran et al. should be further verified [97].

With respect to BIA, it is frequently used in soccer due to the ease of transport (for some models) and speed of use [6]. However, it is probably not the most appropriate method, as the presence of certain diseases, treatments, or clinical situations, as well as the rules of use, can alter the results of this instrument [5,6]. In addition, several published studies point to the existence of a low correlation of FM results between BIA and DXA in professional soccer players, in contrast to some anthropometric formulas [91,99], in agreement with the results of this work. However, the combination of anthropometry and BIA could allow for a more complete assessment of body composition, as BIA adequately assesses water status and cell mass, thus complementing the anthropometric method [84]. Determining water status prior to training and/or competitions could improve hydration patterns and prevent adverse effects such as dehydration and hyperhydration, which have an impact on sports performance or risk of injury [105,106]. Despite their usefulness, the percentage and weight of body water were only collected in a few articles included in the present review, with a value of 47.52 L on average [57,64,66,69,84] (Supplementary Figure S10), as more importance has been traditionally given to the total weight and FM than to the rest of the components [8]. In relation to DXA, although it is considered the gold standard method, it has limitations that can affect the calculations of the measurements, such as the dimensions of the subject to be analyzed (both in height and width), the high economic cost, the experience needed for processing and interpreting the results, or software updates with new algorithms to calculate body composition [6,8,75,107]. In the UEFA expert group statement, the average values of FM of elite male soccer players measured by DXA varied between 8–13%, although lower and higher values have also been reported [2]. Several of the included studies agreed with this range [71,72,73,75,76,78,90], with most of them exceeding it [74,77,79,80,81,82,83,87,88,89,91], but in no case was it below this value. In fact, the mean of the meta-analysis performed provided a value of 13.46%. This does not necessarily translate into poorer performance, as optimal physique varies according to playing position, physiology, and style of play depending on the team and/or coach [2]. Finally, despite being considered the reference method for the evaluation of bone mineral status [7,8], few studies assessed the BMC [75,77,78,79,89], with a value of 3.17 kg (Supplementary Figure S11), or BMD [71,74,75,77,78,79,89] with a value of 1.33 g/cm^3^ (Supplementary Figure S12).

### 4.2. Body Composition Values

Presently, there are multiple body composition references for all professional sports modalities, many of them being considered a goal to achieve for athletes [108,109]. Body composition is crucial for achieving an optimal physical level, which can translate into a good level of play, as performance in soccer depends on multiple technical, biomechanical, tactical, mental, and physiological factors [2,12]. In fact, during soccer practice, there are a multitude of movements that are affected by weight, such as accelerations, changes in direction, or vertical jumps, so muscle training, impact loading, and body fat reduction are important in physical preparation to improve performance in soccer [71,110]. For this reason, the physiological assessment of a soccer player has become more important in recent years, to the extent that it is not only based on technical quality, but also on the physical abilities of the player [111]. Moreover, the physical demands of elite soccer players have been increasing in recent decades, not only in the amount of training and/or competitions, but also in the intensity of effort during matches, and a shorter recovery period between competitions or training sessions [14,112,113]. It is coherent to think that body composition has also evolved over time. However, there are only a few reference values of the different body components in professional soccer players that can be used by the medical technical staff [2,3].

This is not the case in other team sports such as basketball [114] or handball [115], where reference values for some body components, such as FM, are available, although these studies did not specify either the formulas for estimating FM, MM, or BM, or the model of the instrument used. These limitations may influence reliability, reproducibility, and application in clinical practice.

**Figure 6:**
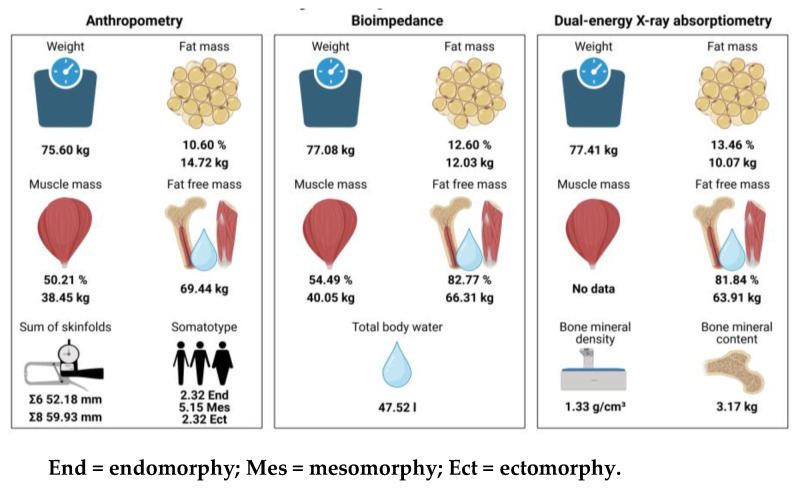
Guidance values for body composition differentiated by measurement method.

In relation to FM, Radzimiński et al. evaluated the relationship between speed, aerobic capacity, body composition (through BIA), and distance covered during official matches, of 23 professional players participating in the international competition Europa League, concluding that players with a lower percentage of FM and higher aerobic capacity covered the longest distances and at a higher speed during competitions [70,116,117]. It has also been observed that a higher percentage of FM is negatively associated with the 20 m sprint speed [118]. Regarding MM, Ayotte et al. assessed whether body mass gain from strength training would impair the aerobic capacity of 11 elite soccer players, with the results showing that it did not negatively impact aerobic capacity, but significantly increased it [2,119,120]. In our work, for the first time to our knowledge, it was possible to make a proposal of ranges of guidance values for the different body compartments (Figure 6).

Recently, Moya-Amaya et al. verified the somatotype trend in the last few decades, of professional male soccer players, observing a decrease in the endomorphic component, evolving from balanced mesomorphy to ecto-mesomorphy [121]. This may be important, because the somatotype that is most prone to injury is the balanced ectomorph (85%), as opposed to the ecto-mesomorph (50%), meso-ectomorph (45%), or balanced mesomorph (44%) [98]. In our study, similar results were observed [22,28,29,30,32,38,44,45,49,85,86], although the total mean endomorphy, ectomorphy, and mesomorphy values were 2.32, 2.32, and 5.15, respectively, resulting in a balanced mesomorph somatotype. These findings relate to the usefulness of the rest of the body compartments, beyond FM, for the performance and health of the soccer player.

### 4.3. Limitations

This study has limitations. Firstly, the existing heterogeneity in the equations for estimating body composition using the anthropometric method, times in the season in which the assessment was carried out, playing positions, and measuring instruments for evaluating the different body components, made it difficult to compare the results. In addition, not all studies that used the anthropometric method provided details on which measurement protocol they applied, so the methodology and anthropometrists could be biased. Moreover, while certain measurement instruments were excluded in the anthropometry section, as they were not valid according to the ISAK protocol, no limiting criteria were applied for BIA or DXA instruments. This is an important aspect, as there is a risk of unifying tetrapolar and octapolar BIA values, as well as single and multi-frequency methods. Another limitation is that although the data collection was limited to male soccer players, not all countries show the same professional level of soccer, with some countries having higher Fédération Internationale de Football Association (FIFA) rankings than others [122]. While it is true that they are all professional players, they do not have the same economic level, sporting facilities, or physical demands, which can affect many factors that could influence their body composition and sporting performance.

In spite of this, and the limitations of our study, it is the only study currently available in the scientific literature that provides a complete description of the main instruments for assessing body composition of male professional soccer players. Based on the current situation of the sport, our research aims to be the first study to propose a range of guidance values of anthropometric measurements, FM, MM, and BM in general male professional soccer players.

### 4.4. Future Research and Practical Application

Although the importance of assessing MM, BM, and body water, and their relationship to sports performance has been highlighted in recent years, studies continue to focus on total weight and FM. For future studies it is recommended to (1) clearly describe the procedures and protocols applied when performing the measurements, (2) specify the reliability, calibration of the measuring instruments, and the technical error of measurement, (3) clearly monitor and report the hydration and nutrition status prior to the measurement, (4) specify the competitive level of the athlete, (5) specify the level of the athlete’s performance, (6) specify the competitive level of the sample by reporting the country and/or region and the name of the league in which the players were competing at the time of the study, (7) report the playing position of the players and the exact time in the season in which the measurements were taken, (8) show all body composition characteristics of the different methods used, as well as the anthropometric and somatotype values, and (9) investigate body composition values through different assessment methods and different playing positions in women’s professional soccer.

Based on the observed results and the experience of the authors, it is proposed (1) to have as many equations and assessment methods as possible; (2) to replace parameters such as “ideal weight” with the sum of skinfolds; (3) to rely on guidance values indicated in the scientific literature, as well as on the evolution of the team and/or players during the season; and finally (4) to work with the medical technical staff and the players and exchange information and impressions.

## 5. Conclusions

This systematic review with meta-analysis provides useful information that could help medical technical staff to adequately assess the body composition of male professional soccer players. In conclusion: (1) the somatotype tendency is balanced mesomorphic; (2) the mean Σ6 and Σ8 skinfolds are 52.18 and 59.93 mm, respectively; (3) there are significant differences in the measurement of the height, percentage, and kilograms of FM, and kilograms of FFM, with oscillating values depending on the method and/or formula applied; and (4) there are no significant differences between measurement methods for the calculation of the weight, percentage of FFM, and percentage and kilograms of MM.

## Figures and Tables

**Table 1:** The inclusion criteria applied in the study followed the Population, Intervention, Comparison, and Outcomes (PICO) strategy.

Population	Intervention	Comparison	Outcomes
Male soccer players who train with the aim of competing or improving their physical performance (excludes physical activity for health or aesthetics). Professional category. Absence of pathologies (healthy subjects).	Anthropometry. Bioimpedance (BIA). Dual X-ray Absorptiometry (DXA).	Measurement methods. Season. Equations.	Anthropometric characteristics: skinfolds, girth, breadth, heights, lengths, body composition, and somatotype. Percentages and values of fat mass, muscle mass, bone mass and body water.

**Table 2:** Descriptive characteristics of the included studies with anthropometry.

Authors and Year	Sample Size (n)	Age (Years)	Country Competition Category	Time of the SEA	Measuring Instruments	Protocol
Al-Hazzaa et al., 2001 [18]	T = 23	25.2 ± 3.3	SA. National team	Preparation for the FR World Cup	BAS Seca, PLI Harpenden	-
Casajús, 2001 [22]	T = 15	25.8 ± 3.1	ES. 1st División	During competitive SEA	EST Rabonne Chesterman, BAS Rabonne Chesterman, Small sliding caliper Rabonne Chesterman, Measuring tape GPM Siber-Hegner Maschinen, PLI Holtain	ISAK
Filaire et al., 2003 [26]	T = 20	25.1 ± 0.4	FR. Ligue 1	Start of pre-SEA, start and end of SEA, start of 2nd pre-SEA	PLI Harpenden	-
Ostojic. 2003 [39]	T = 30	23.5 ± 3.1	EN. National League	Start pre-SEA, start, middle and end SEA, and start 2nd pre-SEA	BAS Avery 3306 ABV, PLI Harpenden	-
Gutierrez and Monroy, 2005 [28]	T = 20	29.0 ± 3.0	MX. National team	Preparation for the World Cup South Korea and Japan	BAS Bame, Swiss anthropometer type Martin, PLI Harpenden	ISAK
Voutselas et al., 2007 [48]	T = 72	20.1 ± 5.2	GR. Super League 1 and 2	-	PLI Harpenden	-
Sotiropoulos et al., 2009 [46]	T = 58; CG = 20; EG = 38	CG = 24.4 ± 2.9; EG = 23.2 ± 2.5	GR. Super League GR	Beginning and end of the transition period	PLI Harpenden	ACSM
Sporis et al., 2009 [47]	T = 270	28.3 ± 5.9	HR. Prva HNL	2 consecutive pre-SEAs	BAS Seca, PLI John Bull Caliper	IBP
Carling and Orhant, 2010 [21]	T = 26	24.4 ± 4.1	FR. Ligue 1	3 consecutive full SEAs (5 moments)	BAS Holtain, PLI Harpenden	-
Dey et al., 2010 [24]	T = 150	23.3 ± 3.5	IN. IN Super League	-	PLI Harpenden	-
Hazir, 2010 [29]	T = 305; SL = 161; FL = 144;	SL = 25.7 ± 3.7 FL = 24.1 ± 4.2	TR. Süper Lig (161) and TFF 1. Lig (144)	5 beginnings of the transition period for the SEA half	BAS Tanita TBF 401A, EST Holtain, Bicondylar caliper Holtain, PLI Holtain	ASRM
Kalapotharakos et al., 2011 [33]	T = 12	25 ± 5	GR. Super League GR	Pre-SEA start, start and mid-SEA	PLI Harpenden	-
Boone et al., 2012 [20]	T = 289	25.4 ± 4.9	BE. Jupiler Pro League	2–4 weeks prior to start of SEA	BAS Seca, PLI Harpenden	-
Chaouachi et al., 2012 [23]	T = 23	19 ± 1	TN. Ligue 1	Last SEA stage	BAS Seca, EST Holtain, PLI Harpenden	-
Owen et al., 2012 [40]	T = 15	24.5 ± 3.4	SCO. Scottish Premiership	During a 4-week break SEA	PLI Harpenden	-
Henríquez-Olguín et al., 2013 [30]	T = 100	23.0 ± 4.4	CL. Not specified	2 SEA starts	BAS Tanita TBF 401A, Kit Health & Performance^®^	ISAK
Jorquera et al., 2013 [32]	T = 406; DEF = 124; CEN = 134; DEL = 93; POR = 48	DEF = 25.3 ± 4; CEN = 25.2 ± 4.7; DEL = 23.5 ± 4.1; POR = 25.1 ± 5.5	CL. 1st división (326) and 1st B (80)	-	BAS Tanita, Kit Gaucho Pro “Mercosur”	ISAK
Lago-Peñas et al., 2013 [34]	T = 42	25.0 ± 5.2	ES. 1st División	During 1st half SEA	PLI Holtain 610	ISAK
Orhan et al., 2013 [38]	1st team = 24 2nd team = 24	23.29 ± 2.12 25.12 ± 3.60	TR. Süper Lig	-	PLI Holtain	ASRM
Iga et al., 2014 [34]	T = 35	20 ± 4	EN. Not specified	Start and end of pre-SEA, 1st and 2nd half and end of SEA	PLI Harpenden	ISAK
Michailidis, 2014 [36]	T = 15	-	GR. Super League Greece	Start and end pre-SEA, middle and end SEA	BAS Tanita BC 418, EST Seca 208, PLI Harpenden	-
Novack et al., 2014 [87]	T = 31	21.48 ± 3.38	BR. Not specified	-	DXA GE Lunar Prodigy software 8.50.093, PLI Harpenden	-
Mascherini et al., 2015 [84]	T = 59	22.47 ± 5.58	IT. Serie A	Pre-SEA start and 50 days later	BAS Akern BIA 101 Sport Edition, Measuring tape Holtain, PLI Holtain	-
Najafi et al., 2015 [37]	T = 60	24.31 ± 4.20	IR. Iran Pro League and Azadegan League	-	BAS Seca, PLI Harpenden	-
Bekris et al., 2016 [19]	T = 24	24.3 ± 4.3	GR. Super League Greece	Start preSEA, start, end 1st half and endSEA	BAS Seca 710, EST Seca, PLI Harpenden	-
Fessi et al., 2016 [25]	T = 17	23.7 ± 3.2	QA. Qatar Stars League	Start and end pre-SEA, and mid-SEA	BAS ADE Electronic Column Scales, EST Holtain, PLI Harpenden	-
Petri et al., 2016 [42]	T = 28	27.88 ± 4.55	IT. Serie A	Pre-SEA start, SEA start and end	BIO BIA 101 Sport, PLI Holtain	-
Lopez-Taylor et al., 2018 [88]	T = 131	23.2 (20.5–26.8)	MX. Liga Premier	-	DXA Hologic QDR4500 Explorer software 12.1, BAS Tanita TBF 410, EST Seca 213, Bicondylar calliper Campbell 10, PLI Harpenden	ISAK
Owen et al., 2018 [41]	T = 22	24.0 ± 3.7	EU. Not specified	Start and end pre-SEA, mid-SEA, end mid-SEA transition period and end SEA	BAS CIRCA, EST CIRCA, Bicondylar calliper Gulick, PLI Harpenden	ISAK
Zuñiga et al., 2018 [49]	T = 78; 1st DIV = 18; 1st “a” DIV = 19; 2nd DIV = 24; 3rd DIV = 17	1st DIV = 25.8 ± 5.2; 1st “a” DIV = 23.4 ± 1.6; 2nd DIV = 18.9 ± 1.6; 3rd DIV = 16.0 ± 0.9	MX. Liga MX (18), Liga de Expansión MX (19), Liga Premier (24) and Liga TDP (17)	Pre-SEA	BAS Tanita Inner Scan BC 532, EST Holtain, Measuring tape Lufkin, Bicondylar caliper Campbell 10, PLI Slimguide	ISAK
Pireva, 2019 [44]	T = 118	-	XK. Superliga	-	BAS Tanita HD-351, EST Seca, PLI John Bull Caliper	-
Rodríguez-Rodríguez et al., 2019 [45]	T = 339; DEF = 119; CEN = 133; DEL = 94; POR = 44	DEF = 25.3 ± 4.8; CEN = 25.2 ± 4.8; DEL = 23.5 ± 4.1; POR = 25.1 ± 5.5	CL. 1st división	-	Kit Gaucho Pro “Mercosur”	ISAK
Campa et al., 2020 [85]	T = 176	Development G = 27.4 ± 4.3; Cross-Validation G = 28.0 ± 5.0	IT. Serie A	-	BAS Seca 877, EST Seca 217, Measuring tape Lufkin, Bicondylar caliper GMP, PLI Holtain	ISAK
Grazioli et al., 2020 [27]	T = 23	26.3 ± 5.6	BR. Brasileirão Serie A	Pre-SEA start and 63 days after quarantine	BAS Urano PP180A, PLI Slimguide	-
Vega et al., 2020 [35]	T = 41	-	ES. 1st División and 2nd División	10 full SEAs	BAS Seca 719, EST Seca 213, PLI Harpenden	ISAK
Pietraszewska et al., 2020 [43]	T = 37	19–30	PL. Ekstraklasa	During competitive SEA	Anthropological instruments Siber Hegner Machinery Ltd., PLI Holtain	ISAK
Castro Jiménez et al., 2021 [86]	T = 24	21.0 ± 1.9	CO. 1st B	-	BIO InBody 770, EST Seca, Bicondylar caliper Holtain, PLI Harpenden HSK-BI	ISAK
Kammerer López et al., 2021 [89]	T = 79	23.0 ± 4.4	CO. 1st A and 1st B	During competitive SEA	DXA GE Lunar Prodigy, BAS Seca 874, EST Seca 213, Measuring tape Lufkin, Bicondylar caliper Slimguide, PLI Harpenden	ISAK

1st = first; 2nd = second; 3rd = third; ACSM = American College of Sports Medicine; ASRM = Anthropometric Standardization Reference Manual; BAS = scale; BE = Belgium; BIO = bioimpedance; BR = Brazil; CEN = midfielders; CG = control group; CL = Chile; CO = Colombia; DEF = defenders; DEL = Forwards; DXA = Dual X-ray absorptiometry; EG = Experimental group; EN = England; ES = Spain; EST = Stadiometer; EU = European players; FR = France; G = Group; GR = Greece; HR = Croatia; IBP = International Biological Program; IN = India; IR = Iran; IT = Italy; ISAK = International Society for the Advancement of Kinanthropometry; MX = Mexico; PL = Poland; PLI = plicometer; POR = goalkeepers; QA = Qatar; SA = Saudi Arabia; SCO = Scotland; T = Total; SEA = Season; TN = Tunisia; TR = Turkey; XK = Kosovo.

**Table 3:** Descriptive characteristics of the included studies with BIA.

Authors and Year	Sample Size (n)	Age (Years)	Country. Competition Category	Time of the SEA	Measuring Instruments
Andreoli et al., 2003 [50]	T = 48; Serie A = 16; Serie B = 14; Serie C = 18	Serie A = 25.9 ± 4.2; Serie B = 25.1 ± 2.6; Serie C = 25.1 ± 5.7	IT. Serie A (16), Serie B (14) and Serie C (18)	-	BAS Invernizzi, EST Invernizzi, Xitron 4000B
Matković et al., 2003 [51]	T = 57	23.2 ± 3.4	HR. Prva HNL	During competitive SEA	Body analyzer Danninger
Dupont et al., 2004 [52]	T = 22	20.2 ± 0.7	FR. Ligue 1	During competitive SEA 1st and 2nd periods	Tanita TBF 543
Al-Jaser and Hasan, 2006 [53]	T = 9	24 ± 4.7	KW. Not specified	5 pre-SEA matches	Biodynamics 310e
Clark et al., 2008 [54]	T = 42	26.0 ± 4.3	EN. Football League Championship	3 complete SEAs (pre-SEA start, mid and end SEA)	EST Seca 240, Tanita TBF 551
Svantesson et al., 2008 [90]	T = 17	24.1 ± 3.8	SE. Allsvenskan	Spring	DXA GE Lunar Prodigy, EST Hultafors, Xitron Hydra 4200
Hoppe et al., 2013 [55]	T = 11	23.8 ± 3.0	DE. Dritte Liga	1st week pre-SEA	Bodystat QuadScan 4000
Suda et al., 2013 [56]	T = 21	24.7 ± 5.2	JP. J2 League	Pre-SEA final, mid-term and SEA final	Tanita MC 190
Micheli et al., 2014 [57]	T = 219	26.1 ± 4.4	IT. Serie A and Serie B	1st half SEA	Akern BIA 101
Mascherini et al., 2015 [84]	T = 59	22.47 ± 5.58	IT. Serie A	Pre-SEA start and 50 days later	Measuring tape Holtain, PLI Holtain, Akern BIA 101 Sport Edition
Semjon et al., 2016 [58]	T = 120; central DEF = 18; full DEF = 15; central CEN = 24; wingers = 18; DEL = 34; POR = 11	n.r.; 27.3 ± 6.2; 26.7 ± 4.8; 25.8 ± 5.3; 25.3 ± 4.2; 24.0 ± 3.6; 26.6 ± 6.5	RC. Českou fotbalovou ligu	6 consecutive pre-SEAs	BAS Leifheit Soehnle 7307, Tanita BC 418 MA
Aras et al., 2017 [59]	T = 12	18.33 ± 0.98	TR. Not specified	-	BAS Jawon Medical
Requena et al., 2017 [60]	T = 19	26.2 ± 2.8	ES. 1ª División	Mid SEA, SEA final and start of pre-SEA	Tanita TBF 543
Kafedžić et al., 2018 [61]	T = 39	23.5 ± 4.6	BA. Premier League	2 pre-SEA starts	Holton Anthropometer, Tanita BC 420SMA
Marcos et al., 2018 [62]	T = 233	25.37 ± 5.06	CY. 1st División	Start pre-SEA	EST Leicester, Tanita BC 418 MA
Suarez-Arrones et al., 2018 [91]	T = 18	27.6 ± 3.0	IT. Serie A	SEA final	DXA Hologic QDR Series Delphi A software 13.3:3, BAS OHAUS, EST Seca 213, Tanita MC-180 MAIII
Clemente et al., 2019 [63]	T = 23	24.7 ± 2.8	PT. 2nd Liga	Pre-SEA start and SEA start	EST Seca 242, Seca mBCA 515
Gardasevic et al., 2019 [64]	T = 70	22.84 ± 4.47	ME. Prva Crnogorska Liga	SEA final	Tanita BC 418 MA
Pietraszewska et al., 2019 [65]	T = 29	25.6 ± 5.8	PL. Ekstraklasa	During competitive SEA	Akern BIA 101 Sport Edition
Campa et al., 2020 [85]	T = 176	Development G = 27.4 ± 4.3; Cross-Validation G = 28.0 ± 5.0	IT. Serie A	-	BAS Seca 877, EST Seca 217, Measuring tape Lufkin, Bicondylar calliper GMP, PLI Holtain, Akern BIA 101
Dağcilar and Öztürk, 2020 [66]	T = 191	24.7 ± 5.5	CY. 1st División	During competitive SEA	Tanita SC 330
Gardasevic and Bjelica, 2020 [67]	T = 53	22.75 ± 4.16	XK. Superliga	SEA final	Tanita BC 418 MA
Granero-Gil et al., 2020 [68]	T = 30	26.57 ± 5.56	RU. Russian Premier League	During competitive SEA	EST Seca, Tanita SC-240
Książek et al., 2020 [69]	T = 26	27.0 ± 3.7	PL. Ekstraklasa	Pre-SEA	Akern
Radzimiński et al., 2020 [70]	T = 23	27.9 ± 4.58	Europa League participants	13 weeks during competitive SEA	Tanita MC-780
Castro Jiménez et al., 2021 [86]	T = 24	21.0 ± 1.9	CO. 1st B	-	EST Seca, Bicondylar calliper Holtain, PLI Harpenden HSK-BI, Tanita BC 418 MA

1st = first; 2nd = second; BA = Bosnia and Herzegovina; BAS = scale; CEN = midfielders; CO = Colombia; CY = Cyprus; DE = Germany; DEF = defenders; DEL = forwards; DXA = dual X-ray absorptiometry; ES = Spain; EST = stadiometer; FR = France; G = group; HR = Croatia; IT = Italy; JP = Japan; KW = Kuwait; ME = Montenegro; PL = Poland; PLI = plicometer; POR = goalkeepers; PT = Portugal; RC = Czech Republic; RU = Russia; SE = Sweden; T = Total; SEA = season; TR = Turkey; XK = Kosovo; n.r. = not reported.

**Table 4:** Descriptive characteristics of the included studies with DXA.

Authors and Year	Sample Size (n)	Age (Years)	Country. Competition Category	Time of the SEA	Measuring Instruments
Wittich et al., 2001 [72]	T = 42	23.2 ± 3.5	AR. 1st División	3 pre-SEAs	GE Lunar DPX-L software 1.33
Svantesson et al., 2008 [90]	T = 17	24.1 ± 3.8	SE. Allsvenskan	Spring	BAS The Advanced Weighing System 31, EST Hultafors, GE Lunar Prodigy
Reinke et al., 2009 [73]	T = 10	25.3 ± 5.1	DE. Bundesliga	SEA final, final of the transitional summer period and final of the pre-SEA	GE Lunar Prodigy software Lunar enCORE 2002
Sutton et al., 2009 [71]	T = 64	26.2 ± 4.0	EN. Premier League	-	BAS y EST Seca 702, Hologic QDR Series Discovery A software 12:4:3
Gerosa-Neto et al., 2014 [74]	T = 82	23.6 ± 4.2	BR. Brasileirão Serie A	Pre-SEA	BAS Filizola, EST Sanny, GE Lunar DPX-MD software 4.7
Novack et al., 2014 [87]	T = 31	21.48 ± 3.38	BR. Not specified	-	PLI Harpenden, GE Lunar Prodigy software 8.50.093
Milanese et al., 2015 [75]	T = 29	27.5 ± 4.38	IT. Serie A	3 full SEAs	BAS Tanita BWB-800MA, EST Harpenden, QDR Explorer W software 12.6.1
Milsom et al., 2015 [76]	T = 27	24.1 ± 3.9	EN. Premier League	3 full SEAs (different periods)	BAS Seca, Hologic QDR Series Discovery A
Sánchez-Ureña et al., 2016 [77]	T = 106	24.53 ± 4.77	CR. Fútbol de 1st División	-	BAS Tanita HD-313, EST Tanita, GE enCORE 2011 software 13.6
Devlin et al., 2017 [78]	T = 18	27 ± 5	AU. A-League	Pre-SEA final	BAS Wedderburn WM203, EST Seca SE206, Hologic Discovery W
Devlin et al., 2017b [79]	T = 18	25 ± 5	AU. A-League	Pre-SEA start, SEA start, SEA middle and SEA final	BAS Wedderburn WM203, EST Seca SE206, Hologic Discovery W
Lopez-Taylor et al., 2018 [88]	T = 131	23.2 (20.5–26.8)	MX. Liga Premier	-	Bicondylar caliper Campbell 10, PLI Harpenden, BAS Tanita TBF 410, EST Seca 213, Hologic QDR4500 Explorer software 12.1
Suarez-Arrones et al., 2018 [91]	T = 18	27.6 ± 3.0	IT. Serie A	SEA final	BAS OHAUS, EST Seca 213, BIO Tanita MC-180 MAIII, Hologic QDR Series Delphi A software 13.3:3
Khalladi et al., 2019 [80]	T = 111	23.7 ± 4.8	QA. Qatar Stars League	During competitive SEA	EST Seca 242, GE Medical SysSEA Lunar software enCORE 12.10
Randell et al., 2019 [81]	T = 16	25 ± 4 26 ± 4	ES. 1st División	2 consecutive pre-SEAs	GE Lunar iDXA
Suarez-Arrones et al., 2019 [82]	T = 10	27.3 ± 2.8	IT. Serie A	SEA final, start and pre-SEA final	BAS OHAUS, EST Seca 213, Hologic QDR Series Delphi A software 13.3:3
McEwan et al., 2020 [83]	T = 20	25.1 ± 4.1	ES. 1st División	Start and end of two pre-SEAs	GE Lunar
Kammerer López et al., 2021 [89]	T = 79	23.0 ± 4.4	CO. 1st A y 1st B	During competitive SEA	Measuring tape Lufkin, Bicondylar caliper Slimguide, PLI Harpenden, BAS Seca 874, EST Seca 213, GE Lunar Prodigy

1st = first; 2nd = second; AR = Argentina; AU = Australia; BAS = Scale; BIO = bioimpedance; BR = Brazil; CO = Colombia; CR = Costa Rica; DE = Germany; EN = England; ES = Spain; EST = stadiometer; IT = Italy; MX = Mexico; PLI = plicometer; QA = Qatar; SE = Sweden; T = Total; SEA = season.

**Table 5:** Analysis of methodological quality according to the Agency for Health Research and Quality (AHRQ) Methodology Checklist.

Authors and Year	1	2	3	4	5	6	7	8	9	10	11	Total *
Al-Hazzaa et al., 2001 [18]	1	0	1	1	0	-	0	0	0	1	0	4
Al-Jaser and Hasan, 2006 [53]	1	0	1	1	0	-	1	1	0	1	1	7
Andreoli et al., 2003 [50]	1	0	0	1	0	-	0	1	0	1	0	4
Aras et al., 2017 [59]	1	0	0	1	0	-	0	0	0	1	0	3
Bekris et al., 2016 [19]	1	0	1	1	0	1	0	1	0	1	1	7
Boone et al., 2012 [20]	1	0	1	1	0	-	0	1	0	1	0	5
Campa et al., 2020 [85]	1	0	0	1	0	-	0	1	0	1	0	4
Carling and Orhant, 2010 [21]	1	0	1	1	0	1	1	1	0	1	1	8
Casajús, 2001 [22]	1	0	1	1	0	1	0	1	0	1	1	7
Castro Jiménez et al., 2021 [86]	1	1	0	1	0	-	0	1	0	1	0	5
Chaouachi et al., 2012 [23]	1	0	1	1	0	-	0	1	0	1	0	5
Clark et al., 2008 [54]	1	1	1	1	0	-	0	1	0	1	1	7
Clemente et al., 2019 [63]	1	1	1	1	0	-	0	1	0	1	1	7
Dağcilar and Öztürk, 2020 [66]	1	0	1	1	0	-	1	0	0	1	0	5
Devlin et al., 2017 [78]	1	0	1	1	0	1	0	1	0	1	0	6
Devlin et al., 2017b [79]	1	0	1	1	0	1	0	1	0	1	1	7
Dey et al., 2010 [24]	1	1	0	1	0	-	0	0	0	1	0	4
Dupont et al., 2004 [52]	1	0	1	1	0	-	0	1	0	1	1	6
Fessi et al., 2016 [25]	1	0	1	1	0	-	1	1	0	1	1	7
Filaire et al., 2003 [26]	1	1	1	1	0	-	0	0	0	1	1	6
Gardasevic and Bjelica, 2020 [67]	1	0	1	1	0	-	0	0	0	1	0	4
Gardasevic et al., 2019 [64]	1	0	1	1	0	-	0	0	0	1	0	4
Gerosa-Neto et al., 2014 [74]	1	0	1	1	0	-	0	0	0	1	0	4
Granero-Gil et al., 2020 [68]	1	1	1	1	0	-	0	0	0	1	0	5
Grazioli et al., 2020 [27]	1	0	1	1	0	-	0	1	0	1	1	6
Gutierrez and Monroy, 2005 [28]	1	0	1	1	0	1	0	1	0	1	0	6
Hazir, 2010 [29]	1	0	1	1	0	-	0	1	0	1	-	5
Henríquez-Olguín et al., 2013 [30]	1	0	1	1	0	-	0	0	0	1	1	5
Hoppe et al., 2013 [55]	1	0	1	1	0	-	0	1	0	1	0	5
Iga et al., 2014 [34]	1	0	1	1	0	-	0	1	0	1	1	6
Jorquera et al., 2013 [32]	1	0	0	1	0	-	0	1	0	1	0	4
Kafedžić et al., 2018 [61]	1	0	1	1	0	-	0	1	0	1	1	6
Kalapotharakos et al., 2011 [33]	1	0	1	1	0	-	0	0	0	1	1	5
Kammerer López et al., 2021 [89]	1	1	1	1	0	1	0	1	0	1	0	7
Khalladi et al., 2019 [80]	1	1	1	1	0	-	1	0	0	1	0	6
Książek et al., 2020 [69]	1	1	1	1	0	-	0	1	0	1	0	6
Lago-Peñas et al., 2013 [34]	1	1	1	1	0	-	0	1	0	1	0	6
Lopez-Taylor et al., 2018 [88]	1	0	0	1	0	1	0	1	0	1	1	6
Marcos et al., 2018 [62]	1	1	1	1	0	-	0	1	0	1	0	6
Mascherini et al., 2015 [84]	1	1	1	1	0	-	1	1	0	1	1	8
Matković et al., 2003 [51]	1	0	1	1	0	-	0	0	0	1	0	4
McEwan et al., 2020 [83]	1	0	1	1	0	-	0	1	0	1	1	6
Michailidis, 2014 [36]	1	0	1	1	0	-	1	1	0	1	1	7
Micheli et al., 2014 [57]	1	0	1	1	0	-	0	1	0	1	0	5
Milanese et al., 2015 [75]	1	0	1	1	0	1	1	1	0	1	1	8
Milsom et al., 2015 [76]	1	0	1	1	0	1	0	1	0	1	1	7
Najafi et al., 2015 [37]	1	0	0	1	0	-	0	0	0	1	0	3
Novack et al., 2014 [87]	1	1	0	1	0	1	0	1	0	1	0	6
Orhan et al., 2013 [38]	1	0	0	1	0	-	0	1	0	1	0	4
Ostojic. 2003 [39]	1	0	1	1	0	-	0	0	0	1	1	5
Owen et al., 2012 [40]	1	0	1	1	0	-	0	1	0	1	0	5
Owen et al., 2018 [41]	1	0	1	1	0	1	0	1	0	1	1	7
Petri et al., 2016 [42]	1	0	1	1	0	-	0	1	0	1	1	6
Pietraszewska et al., 2019 [65]	1	0	1	1	0	-	0	1	0	1	0	5
Pietraszewska et al., 2020 [43]	1	1	0	1	0	-	0	1	0	1	0	5
Pireva, 2019 [44]	1	1	0	1	0	-	0	0	0	1	0	4
Radzimiński et al., 2020 [70]	1	1	1	1	0	-	0	1	0	1	1	7
Randell et al., 2019 [81]	1	0	1	1	0	-	0	1	0	1	1	6
Reinke et al., 2009 [73]	1	0	1	1	0	-	0	0	0	1	1	5
Requena et al., 2017 [60]	1	0	1	1	0	-	1	1	0	1	1	7
Rodríguez-Rodríguez et al., 2019 [45]	1	1	0	1	0	1	0	1	0	1	0	6
Sánchez-Ureña et al., 2016 [77]	1	0	0	1	0	-	0	1	0	1	0	4
Semjon et al., 2016 [58]	1	1	1	1	0	-	1	0	0	1	1	8
Sotiropoulos et al., 2009 [46]	1	1	1	1	0	-	0	1	0	1	0	6
Sporis et al., 2009 [47]	1	1	1	1	0	-	0	1	0	1	1	7
Suarez-Arrones et al., 2018 [91]	1	0	1	1	0	1	0	1	0	1	0	6
Suarez-Arrones et al., 2019 [82]	1	1	1	1	0	1	0	1	0	1	1	8
Suda et al., 2013 [56]	1	0	1	1	0	-	0	1	0	1	1	6
Sutton et al., 2009 [71]	1	0	0	1	0	-	0	1	0	1	0	4
Svantesson et al., 2008 [90]	1	0	1	1	0	1	0	1	0	1	0	6
Vega et al., 2020 [35]	1	1	1	1	0	-	0	1	0	1	1	7
Voutselas et al., 2007 [48]	1	0	0	1	0	-	0	1	0	1	0	4
Wittich et al., 2001 [72]	1	0	1	1	0	-	1	0	1	1	1	7
Zuñiga et al., 2018 [49]	1	0	1	1	0	-	0	1	0	1	0	5

* 0 = worst; 11 = best value; - = unclear.

**Table 6:** Meta-analysis by groups according to the evaluation method used.

	Authors	G	M	CI 95%	Weight (%)	M	CI95%	*p*
Age								
Anthropometry	[18,19,20,21,22,23,24,25,26,27,28,29,30,31,32,33,34,37,38,39,40,41,42,43,45,46,47,48,49,84,85,86,87,88,89]	48	24.20	23.45; 24.95	49.3	24.50	24.04; 24.97	0.45
BIA	[50,51,52,53,54,55,56,57,58,59,60,61,62,63,64,65,66,67,68,69,70,84,85,86,90,91]	35	24.79	24.02; 25.56	34.1			
DXA	[71,72,73,74,75,76,77,78,79,80,81,82,83,87,88,89,90,91]	17	24.78	23.90; 25.67	16.7			
Height								
Anthropometry	[18,19,20,21,22,23,24,25,26,28,29,30,31,32,33,34,35,37,38,39,40,41,42,43,44,45,46,47,48,49,84,85,86,87,88,89]	120	179.01	178.32; 179.70	58.4	179.76	179.22; 180.30	<0.01
BIA	[50,51,52,53,54,55,56,57,58,59,60,61,62,63,64,65,66,67,68,69,70,84,85,86,90,91]	52	181.17	180.17; 182.16	24.1			
DXA	[71,72,73,74,75,76,77,78,79,80,81,82,83,87,88,89,90,91]	38	180.37	178.91; 181.83	17.5			
Weight								
Anthropometry	[18,19,20,21,22,23,24,25,26,27,28,29,30,31,32,33,34,35,36,37,38,39,40,41,42,43,44,45,46,47,48,49,84,85,86,87,88,89]	70	75.60	74.57; 76.62	56.8	76.27	75.51; 77.03	0.11
BIA	[50,51,52,53,54,55,56,57,58,59,60,61,62,63,64,65,66,67,68,69,70,84,85,86,90,91]	38	77.08	75.88; 78.27	30.5			
DXA	[71,72,73,74,75,76,77,78,79,80,81,82,83,87,88,89,90,91]	16	77.41	74.41; 80.19	12.7			
Fat mass kilograms								
Anthropometry	[34,41,44,45,89]	14	14.72	12.82; 16.61	40.7	12.48	11.41; 13.55	<0.01
BIA	[50,56,57,65,69,85]	9	12.03	10.41; 13.66	25.6			
DXA	[72,73,74,75,76,78,79,81,82,83,87,89]	12	10.07	9.35; 10.79	33.7			
Fat mass percentage								
Anthropometry	[18,19,20,21,22,23,24,25,26,27,28,31,33,34,35,36,37,39,44,46,47,48,87,88,89]	44	10.60	9.73; 11.47	44.8	11.85	11.28; 12.43	<0.01
BIA	[50,51,52,53,54,55,56,57,58,59,60,61,62,63,64,65,66,67,68,69,70,85,86,90,91]	40	12.60	11.76; 13.44	38.4			
DXA	[71,72,73,74,75,76,77,78,79,80,81,82,83,87,88,89,90,91]	17	13.46	12.20; 14.73	16.8			
Fat-Free mass kilograms								
Anthropometry	[21,22,36,39,41,42,44]	9	69.44	67.56; 71.32	22.9	66.10	64.65; 67.55	<0.01
BIA	[50,51,56,57,65,66,69,84,85,90]	13	66.31	64.67; 67.96	37.2			
DXA	[72,73,74,75,76,77,80,81,82,83,87,89,90]	14	63.91	61.09; 66.74	39.9			
Fat-Free mass percentage								
BIA	[63,65,69]	3	82.77	75.55; 89.98	48.9	82.36	80.30; 84.42	0.59
DXA	[71,72,89]	3	81.84	79.84; 83.81	51.1			
Muscle mass kilograms								
Anthropometry	[34,44,45,89]	7	38.45	36.17; 40.73	48.7	39.28	37.34; 41.23	0.37
BIA	[64,65,67,69,86]	8	40.05	36.46; 43.64	51.3			
Muscle mass percentage								
Anthropometry	[28,34,44,89]	4	50.21	45.45; 54.97	57.3	52.03	46.90; 57.17	0.38
BIA	[61,65,69]	3	54.49	34.67; 74.31	42.7			

G = groups; M = mean.

**Table 7:** Meta-analysis by groups according to the equation used.

	Authors	G	M	CI 95%	Weight (%)	M	CI95%	*p*
Fat mass								
Equation (1)	[22,89]	3	8.19	7.24; 9.15	8.5	10.19	9.41; 10.97	<0.01
Equation (3)	[19,21,23,24,25,26,31,48,87,89]	13	12.18	11.06; 13.30	35.9			
Equation (6)	[27,34,37,89]	4	11.16	10.42; 11.9	11.3			
Equation (9)	[36,39,46,47,87,89]	6	9.54	7.45; 11.62	16.8			
Equation (11)	[33,87]	2	9.23	−6.33; 24.78	5.3			
Equation (20)	[31,88,89]	2	10.02	9.77; 10.27	5.7			
Equation (26)	[87,88]	6	7.14	6.87; 7.41	16.6			
Sum of skinfold								
Equation (5)	[22,32,34,45]	11	52.18	49.49; 54.87	78.4	53.95	51.19; 56.70	<0.01
Equation (6)	[31,40,41]	4	59. 93	55.34; 64.52	21.6			

Fat mass: Equation (1) = Carter, 1982; Equation (3) = Durnin and Womersley, 1974; Equation (6) = Faulkner, 1968; Equation (9) = Jackson and Pollock 7 folds, 1978; Equation (11) = Jackson and Pollock 3 folds, 1978; Equation (20) = Reilly, 2009; Equation (26) = Yuhasz, 1974. Sum of skinfolds: Equation (5) = Σ6 skinfolds (triceps, subscapular, supraspinale, abdominal, mid-thigh, and calf); Equation (6) = Σ8 folds (biceps, triceps, subscapular, iliac crest, supraspinale, abdominal, mid-thigh, and calf). G = groups; M = mean.

## Data Availability

The data presented in this study are available in the tables of this article. The data presented in this study are available on request from the corresponding author.

## Supplementary Materials

The following supporting information can be downloaded at: https://www.mdpi.com/article/10.3390/nu15051160/s1, Table S1: Body composition characteristics of included studies with anthropometry; Table S2: Body composition characteristics of included studies with BIA; Table S3. Body composition characteristics of included studies with DXA; Figure S1. Forest plot of age according to assessment method; Figure S2. Forest plot of height according to assessment method; Figure S3. Forest plot of weight according to assessment method; Figure S4. Forest plot of fat mass kilograms according to assessment method; Figure S5. Forest plot of fat mass percentage according to assessment method; Figure S6. Forest plot of fat-free mass kilograms according to assessment method; Figure S7. Forest plot of fat-free mass percentage according to assessment method; Figure S8. Forest plot of muscle mass kilograms according to assessment method; Figure S9. Forest plot of muscle mass percentage according to assessment method; Figure S10. Forest plot of total body water; Figure S11. Forest plot of bone mineral content; Figure S12. Forest plot of bone mineral density

## Abbreviations

ACSM: American College of Sports Medicine; AHRQ: Agency for Health Research and Quality; ASRM: Anthropometric Standardization Reference Manual; BIA: bioelectrical impedance analysis; BM: bone mass; BMC: bone mineral content; BMD: bone mineral density; DXA: dual X-ray absorptiometry; FIFA: Fédération Internationale de Football Association; FFM: fat-free mass; FM: fat mass; IBP: International Biological Program; ISAK: International Society for the Advancement of Kinanthropometry; MM: muscle mass; PICO: Population/Intervention/Comparison/Outcomes; PRISMA: Preferred Reporting Items for Systematic Reviews and Meta-Analyses; REML: restricted maximum likelihood.
