# Supplementary material for: Body Composition of Male Professional Soccer Players Using Different Measurement Methods: A Systematic Review and Meta-Analysis
Source: Nutrients. 2023 Feb 25;15(5):1160. doi: 10.3390/nu15051160 (PMC10005265; doi:10.3390/nu15051160)
Supplement: Supplementary file 1 [file nutrients-15-01160-s001.zip › Tables S1-S3.pdf]

Table S1

Authors and years	Sample size (n)	Time of the SEA	Age (years)	Height (cm)	Weight (kg)	Sum of skinfold (mm)	Fat mass (%)	Fat mass (kg)	Muscle mass (%)	Muscle mass (kg)	Fat-free mass (kg)	Endomorphy	Ectomorphy	Mesomorphy
Al-Hazzaa et al., 2001	Total (23)	Preparation for the World cup	25.2 ± 3.3	177.2 ± 5.9	73.1 ± 6.8	14.2 ± 2.6[2]	12.3 ± 2.7[L2]	-	-	-	-	-	-	-
Casajús, 2001	Total (15)	1st measurement	25.8 ± 3.1	180 ± 7	78.6 ± 6.6	57.0 ± 8.6[6]	8.6 ± 0.91[C]	-	-	-	71.9 ± 6.0	2.6 ± 0.5	2.3 ± 0.7	4.9 ± 0.8
		2nd measurement	26.3 ± 3.1	180 ± 8	78.5 ± 6.4	52.9 ± 8.6[6]	8.2 ± 0.91[C]	-	-	-	72.1 ± 5.7	2.4 ± 0.5	2.3 ± 0.7	4.8 ± 0.8
Filaire et al., 2003	Total (20)	Start of pre-SEA	25.1 ± 0.4	181.5 ± 1.4	78.3 ± 1.5	-	12.3 ± 0.9[D]	-	-	-	-	-	-	-
Ostojic, 2003	Total (30)	Start pre-SEA	23.5 ± 3.1	182.8 ± 6.0	77.8 ± 6.3	-	11.5 ± 2.1[J7]	-	-	-	68.8 ± 5.3	-	-	-
Gutierrez & Monroy, 2005	Total (20)	Preparation for the World cup	29.0 ± 3.0	175.0 ± 5.6	74.0 ± 5.6	-	10.0 ± 1.6[J7b]	-	49.0 ± 1.3[M]	-	-	2.35 ± 0.48	1.95 ± 0.77	5.9 ± 0.9
Voutselas et al., 2007	Total (72)	-	20.1 ± 5.2	173.4 ± 22.0	70.2 ± 12.3	-	8.5 ± 4.6[D]	-	-	-	-	-	-	-
Sotiropoulos et al., 2009	Total (58)	Beginning of the transition period	24.4 ± 2.9	178.6 ± 4.1	77.57 ± 4.21	-	7.87 ± 1.70[J7]	-	-	-	-	-	-	-
Sporis et al., 2009	Total (270)	2 consecutive pre-SEAs	28.3 ± 5.9	181.4 ± 2.5	78.4 ± 3.1	-	11.9 ± 3.1[J7]	-	-	-	-	-	-	-
Carling & Orhant, 2010	Total (26)	Start of pre-SEA	24.4 ± 4.1	182.1 ± 5.8	77.48 ± 6.04	-	10.81 ± 1.77[D]	-	-	-	69.11 ± 5.43	-	-	-
Dey et al., 2010	Total (150)	-	23.3 ± 3.5	-	-	-	-	-	-	-	-	-	-	-
	Defenders (44)		23.1 ± 3.2	170.8 ± 5.7	63.2 ± 8.0	-	13.8 ± 2.0[D]	-	-	-	-	-	-	-
	Midfielders (48)		23.3 ± 3.6	171.9 ± 5.9	64.9 ± 6.5	-	13.3 ± 2.4[D]	-	-	-	-	-	-	-
	Forwards (35)		23.2 ± 3.4	170.9 ± 5.7	63.5 ± 6.3	-	13.6 ± 2.1[D]	-	-	-	-	-	-	-
	Goalkeepers (23)		23.3 ± 3.9	173.8 ± 5.3	66.7 ± 5.5	-	14.0 ± 2.6[D]	-	-	-	-	-	-	-

Hazir, 2010	Total (305)	5 beginnings of the transition period for the SEA half	-	-	-	-	-	-	-	-	-	-	-	-
	(161 SL)		25.7 ± 3.7	178.4 ± 5.6	76.1 ± 6.1	-	-	-	-	-	-	2.5 ± 0.7	2.3 ± 0.7	4.8 ± 0.9
	(144 FL)		24.1 ± 4.2	178.4 ± 5.9	73.9 ± 6.3	-	-	-	-	-	-	3.0 ± 0.9	2.6 ± 0.8	4.5 ± 0.9
Kalapothar akos et al., 2011	Total (12)	Start of pre-SEA	25 ± 5	179 ± 6	74.2 ± 6.5	-	10.5 ± 2.5[J3]	-	-	-	-	-	-	-
Boone et al., 2012	Total (289)	Pre-SEA	25.4 ± 4.9	182.4 ± 6.0	77.4 ± 7.1	-	11.0 ± 2.5[P]	-	-	-	-	-	-	-
Chaouachi et al., 2012	Total (23)	End of SEA	19 ± 1	181.0 ± 5.7	73.2 ± 4.1	-	11.0 ± 2.4[D]	-	-	-	-	-	-	-
Owen et al., 2012	Total (15)	1st measurement	24.5 ± 3.4	181.1 ± 5.7	78.7 ± 7.6	60.23 ± 16.21[8]	-	-	-	-	-	-	-	-
		2nd measurement				59.23 ± 14.78[8]								
Henríquez-Olguín et al., 2013	Total (100)	2 consecutive start of SEA	23.0 ± 4.4	-	-							-	-	-
	Central defenders (17)		24.8 ± 4.0	181.4 ± 4.2	78.2 ± 4.5	-	-	-	-	-	-	2.23 ± 0.74	2.48 ± 0.57	5.29 ± 0.68
	Lateral defenders (14)		23.2 ± 2.6	174.6 ± 5.7	73.0 ± 5.7	-	-	-	-	-	-	2.27 ± 0.49	2.01 ± 0.52	5.52 ± 0.99
	Deffensive midfielders (18)		23.8 ± 4.9	175.6 ± 5.8	71.6 ± 6.0	-	-	-	-	-	-	2.16 ± 0.59	2.42 ± 0.83	5.14 ± 0.93
	Offensive midfielders (17)		23.3 ± 3.8	172.4 ± 5.2	72.2 ± 5.4	-	-	-	-	-	-	2.36 ± 0.67	1.76 ± 0.71	5.51 ± 1.07
	Forwards (23)		23.2 ± 4.5	177.1 ± 5.4	73.7 ± 7.1	-	-	-	-	-	-	2.14 ± 0.67	2.39 ± 0.70	5.12 ± 0.79
	Goalkeepers (11)		23.0 ± 4.4	184.0 ± 2.8	81.3 ± 0.5	-	-	-	-	-	-	2.35 ± 0.79	2.53 ± 0.51	5.31 ± 0.85
Jorquera et al., 2013	Total (406)	-	-	-	-	-	-	-	-	-	-	-	-	-
	Defenders (124)		25.3 ± 4.8	178.1 ± 5.5	77.1 ± 6.2	51.3 ± 13.6[6]	-	-	-	-	-	2.25	2.10	5.40
	Midfielders (134)		25.2 ± 4.7	172.9 ± 5.6	71.7 ± 6.0	50.5 ± 11.6[6]	-	-	-	-	-	2.20	1.94	5.51
	Forwards (93)		23.5 ± 4.1	176.6 ± 6.1	75.2 ± 7.2	48.8 ± 11.5[6]	-	-	-	-	-	2.13	2.10	5.32
	Goalkeepers (48)		25.1 ± 5.5	181.2 ± 3.8	81.4 ± 5.9	58.9 ± 12.0[6]	-	-	-	-	-	2.53	2.05	5.49
Lago-Peñas et al., 2013	Total (42)	Start of pre-SEA	25.0 ± 5.2	177.3 ± 6.7	75.72 ± 6.37	47.45 ± 7.70[6]	10.99 ± 1.02[F]	8.34 ± 1.27	47.96 ± 0.87[M]	36.33 ± 3.12	-	-	-	-

Orhan et al., 2013	1st team (24)<br>2nd team (24)	-	23.29 ± 2.12<br>25.12 ± 3.60	179.28 ± 5.71<br>179.08 ± 5.73	76.86 ± 5.39<br>76.60 ± 6.56	-<br>-	-<br>-	-<br>-	-<br>-	-<br>-	-<br>-	2.28 ± 0.41<br>2.34 ± 0.44	2.31 ± 0.64<br>2.30 ± 0.61	4.40 ± 1.05<br>4.35 ± 0.93
Iga et al., 2014	Total (35)	Start of pre-SEA	20 ± 4	182 ± 6	77.1 ± 7.3	26.9 ± 4.3[D]<br>32.4 ± 7.5[R]<br>35.6 ± 6.1[5]<br>57.5 ± 11.3[8]	12.2 ± 1.9[D]<br>10.0 ± 1.1[R]	-	-	-	-	-	-	-
Michailidis, 2014	Total (15)	Start of pre-SEA	-	-	76.70 ± 1.82	-	10.32 ± 0.73[J7]	-	-	-	68.76 ± 1.65	-	-	-
Novack et al., 2014	Total (31)	-	21.48 ± 3.38	181.97 ± 8.11	79.05 ± 9.48	-	15.09 ± 3.47[D]<br>8.38 ± 2.80[J7]<br>8.05 ± 3.02[J3]<br>7.48 ± 1.01[L1]<br>10.49 ± 2.46[W]	-	-	-	-	-	-	-
Mascherini et al., 2015	Total (59)	Start of pre-SEA	22.47 ± 5.58	181 ± 8	76.48 ± 8.35	56.17 ± 12.25[8b]	-	-	-	-	-	-	-	-
Najafi et al., 2015	Total (60)	-	24.31 ± 4.20	179 ± 6	75.40 ± 7.14	-	11.08 ± 1.50[F]	-	-	-	-	-	-	-
Bekris et al., 2016	Total (24)	Start of pre-SEA	24.3 ± 4.3	180.3 ± 3.8	77.44 ± 6.13	-	10.33 ± 1.59[D]	-	-	-	-	-	-	-
Fessi et al., 2016	Total (17)	Start of pre-SEA	23.7 ± 3.2	177.9 ± 6.0	72.9 ± 8.4	-	10.4 ± 1.9[D]	-	-	-	-	-	-	-
Petri et al., 2016	Total (28)	Start of pre-SEA	27.88 ± 4.55	184 ± 6	79.9 ± 6.2	-	-	-	-	-	72.5 ± 5.1	-	-	-
Lopez-Taylor et al., 2018	Total (131)	-	23.2 (20.5 – 26.8)	177.5 ± 5.8	73.2 ± 8.0	-	9.2 (6.3–12.0)[T]<br>9.4 (7.4–11.3)[Wh]<br>10.2 (7.2–13.3)[S]<br>10.5 (8.6–13.1)[W]	-	-	-	-	-	-	-

							10.5 (8.9–12.7)[E]<br>10.5 (9.3–12.2)[Pa]<br>10.7 (9.6–12.2)[R]<br>10.8 (7.8–14.4)[Tb]<br>11.2 (9.4–13.5)[Eb]<br>12.6 (10.5–14.5)[Ci]<br>13.0 (9.6–17.9)[Fo4]<br>13.1 (11.0–15.5)[O]<br>13.7 (10.1–18.4)[Fo2]<br>14.3 (12.5–17.6)[Z]							
Owen et al., 2018	Total (22)	Start of pre-SEA	24.0 ± 3.7	180.45 ± 5.12	76.66 ± 5.34	63.51 ± 11.87[8]	-	7.47 ± 1.78	-	-	67.21 ± 5.31	-	-	-
Zuñiga et al., 2018	Total (78)	Pre-SEA	-	-	-	-	-	-	-	-	-	-	-	-
	1ª división (18)		25.8 ± 5.2	175.1 ± 5.6	75.1 ± 7.2	-	-	-	-	-	-	2.3 ± 0.8	1.9 ± 1.0	5.7 ± 1.1
	1ª “a” división (19)		23.4 ± 1.6	175.0 ± 4.8	72.9 ± 4.4	-	-	-	-	-	-	2.5 ± 0.6	2.1 ± 0.6	5.3 ± 0.7
	2ª división (24)		18.9 ± 1.6	175.0 ± 5.6	75.1 ± 7.2	-	-	-	-	-	-	2.6 ± 1.0	3.1 ± 1.0	4.3 ± 0.8
	3ª división (17)		16.0 ± 0.9	169.4 ± 4.0	60.9 ± 6.8	-	-	-	-	-	-	2.3 ± 0.8	3.1 ± 1.3	4.4 ± 1.4
Pireva, 2019	Total (118)	-	-	180.78 ± 6.06	77.91 ± 6.63	-	14.43 ± 1.42[M]	10.92 ± 3.24	54.62 ± 3.38[M]	42.62 ± 5.03	66.65 ± 5.52	2.4 ± 0.7	2.4 ± 0.7	5.4 ± 1.0
Rodríguez-Rodríguez et al., 2019	Total (339)	-	-	-	-	-	-	-	-	-	-	-	-	-
	Defenders (119)		25.3 ± 4.8	178.2 ± 5.6	77.1 ± 6.2	51.4 ± 13.7[6]	-	16.2 ± 2.6	-	38.9 ± 3.6	-	2.3 ± 0.7	2.1 ± 0.7	5.4 ± 0.9
	Midfielders (133)		25.2 ± 4.8	172.9 ± 5.6	71.7 ± 6.0	50.6 ± 11.6[6]	-	15.0 ± 2.0	-	35.8 ± 3.6	-	2.3 ± 0.6	1.9 ± 0.8	5.5 ± 1.0
	Forwards (94)		23.5 ± 4.1	176.6 ± 6.2	75.2 ± 7.2	48.8 ± 11.5[6]	-	15.5 ± 2.4	-	37.8 ± 5.1	-	2.1 ± 0.6	2.1 ± 0.7	5.3 ± 1.1
	Goalkeepers (44)		25.1 ± 5.5	181.3 ± 3.8	81.4 ± 5.9	58.9 ± 12.0[6]	-	18.0 ± 2.1	-	40.7 ± 4.1	-	2.5 ± 0.5	2.1 ± 0.7	5.5 ± 0.9
Campa et al., 2020	Total (176)	-	27.4 ± 4.3[GD]	183.8 ± 0.5[GD]	79.5 ± 6.1[GD]	-	-	-	-	-	-	2.0 ± 0.4[GD]	2.7 ± 0.6[GD]	4.9 ± 0.8[GD]
			28.0 ± 5.0[GV]	182.9 ± 0.5[GV]	78.9 ± 6.5[GV]	-	-	-	-	-	-	1.9 ± 0.4[GV]	2.7 ± 0.6[GV]	5.2 ± 0.8[GV]

Grazioli et al., 2020	Total (23)	Start of pre-SEA	26.3 ± 5.6	-	78.43 ± 8.70	-	10.67 ± 1.71[F]	-	-	-	-	-	-	-
Vega et al., 2020	Total (41)	10 consecutive SEA	-	-	-	-	-	-	-	-	-	-	-	-
	Central defenders (5)		-	184 ± 3	79.46 ± 2.79	-	7.34 ± 1.13[Y]	-	-	-	-	-	-	-
	Lateral defenders (10)		-	176 ± 4	71.63 ± 3.92	-	7.11 ± 0.94[Y]	-	-	-	-	-	-	-
	Central midfielders (11)		-	178 ± 5	73.77 ± 6.24	-	7.01 ± 0.65[Y]	-	-	-	-	-	-	-
	Lateral midfielders (5)		-	179 ± 4	71.97 ± 3.19	-	7.07 ± 0.70[Y]	-	-	-	-	-	-	-
	Forwards (7)		-	185 ± 5	78.82 ± 6.44	-	7.23 ± 1.32[Y]	-	-	-	-	-	-	-
	Goalkeepers (3)		-	190 ± 2	84.03 ± 1.20	-	7.91 ± 0.83[Y]	-	-	-	-	-	-	-
Pietraszewska et al., 2020	Total (37)	During competitive SEA	19-30	181.6 ± 5.5	76.8 ± 7.2	-	-	-	-	-	-	-	-	-
Castro Jiménez et al., 2021	Total (24)	-	21.0 ± 1.9	177.3 ± 4.3	73.21 ± 6.42	-	-	-	-	-	-	-	-	-
	Defenders (5)		-	181.7 ± 6.1	75.3 ± 8.3	-	-	-	-	-	-	2.08 ± 0.8	2.78 ± 0.7	5.0 ± 0.3
	Midfielders (13)		-	175 ± 4.6	69.7 ± 4.3	-	-	-	-	-	-	2.13 ± 0.7	2.7 ± 0.7	5.3 ± 0.9
	Forwards (4)		-	174.3 ± 4.0	76.4 ± 5.5	-	-	-	-	-	-	2.3 ± 0.8	1.58 ± 0.5	6.8 ± 0.8
	Goalkeepers (2)		-	185.2 ± 5.2	80.9 ± 0.8	-	-	-	-	-	-	1.7 ± 0.6	2.8 ± 1.0	5.85 ± 1.1
Kammerer López et al., 2021	Total (79)	During competitive SEA	23.0 ± 4.4	178.4 ± 6.8	75.2 ± 7.7	-	7.20 ± 2.58[J7]<br>7.84 ± 1.56[C]<br>10.04 ± 1.43[R]<br>11.82 ± 2.17[F]<br>11.93 ± 2.88[PB]<br>13.05 ± 3.63[D]<br>21.83 ± 3.14[K]	16.37 ± 2.61	49.27 ± 3.02[K]	37.10 ± 4.97	-	-	-	-

C=Carter, 1982; Ci=Civar, 2003; D=Durnin y Womersley, 1974; E=Evans 3 pliegues, 2005; Eb=Evans 7 pliegues, 2005; F=Faulkner, 1968; Fo4=Forsyth 4 pliegues, 1973; Fo2=Forsyth 2 pliegues, 1973; GD=Development group; GV=Validation group; J7=Jackson y Pollock 7 pliegues, 1978; J7b=Jackson y Pollock 7 pliegues, 1985; J3=Jackson y Pollock 3 pliegues, 1978; K=Kerr, 1988; L1=Lohman, 1981; L2=Lohman, 1992; M=Matiegka, 1921; O=Oliver, 2012; P=Parizkova, 1977; PB=Pariskova y Buskova, 1971; Pa=Pascale, 1956; R=Reilly, 2009; S=Stewart, 2000; SEA=Season; T=Thorland 3 pliegues, 1984; Tb=Thorland 7 pliegues, 1984; W=Withers et al., 1987; Wh=White, 1980; Y=Yuhasz, 1974; Z=Zuti, 1973; 2=Sum of 2 skinfold (triceps and subscapular); 4D=Sum of 4 skinfold Durnin y Womersley, 1974 (triceps, subscapular, iliac crest and supraspinale); 4R=Sum of 4 skinfold Reilly, 2009 (triceps, abdominal, mid-thigh and calf); 5=Sum

of 5 skinfold (biceps, triceps, subscapular, iliac crest, supraspinale, mid-thigh); 6=Sum of 6 skinfold (triceps, subscapular, supraspinale, abdominal, mid-thigh, and calf); 8=Sum of 8 skinfold (biceps, triceps, subscapular, iliac crest, supraspinale, abdominal, mid-thigh, and calf); 8b=Sum of 8 skinfold (triceps, biceps, subscapularis, iliac crest, mid-armpit, pectoral, abdominal and mid-thigh)

Table S2

Authors and years	Sample size (n)	Time of the SEA	Age (years)	Height (cm)	Weight (kg)	Fat mass (%)	Fat mass (kg)	Muscle mass (%)	Muscle mass (kg)	Fat-free mass (%)	Fat-free mass (kg)	Total body water (l)
Andreoli et al., 2003	Total (48)	-	-	-	-	-	-	-	-	-	-	-
	Serie A (16)		25.9 ± 4.2	183.3 ± 6.7	81.6 ± 6.1	15.4 ± 3.6	13.3 ± 2.8	-	-	-	68.2 ± 5.8	-
	Serie B (14)		25.1 ± 2.6	182.6 ± 3.8	77.7 ± 5.7	15.8 ± 2.7	12.3 ± 2.3	-	-	-	65.4 ± 5.1	-
	Serie C (18)		25.1 ± 5.7	180.9 ± 5.6	73.7 ± 8.2	13.9 ± 4.8	10.5 ± 4.0	-	-	-	63.2 ± 5.0	-
Matković et al., 2003	Total (57)	During competitive SEA	23.2 ± 3.4	180.6 ± 5.7	77.6 ± 5.7	14.9 ± 3.5	-	-	-	-	66.3 ± 5.4	-
Dupont et al., 2004	Total (22)	1st measurement	20.2 ± 0.7	178.0 ± 4.9	71.3 ± 5.7	14.7 ± 2.4	-	-	-	-	-	-
		2nd measurement			71.8 ± 6.2	15.0 ± 2.6	-	-	-	-	-	-
		3rd measurement			71.5 ± 5.9	14.6 ± 2.3	-	-	-	-	-	-
Al-Jaser & Hasan, 2006	Total (9)	Pre-SEA	24 ± 4.7	173.4 ± 5.2	68.2 ± 7.0	12.3 ± 3.7	-	-	-	-	-	-
Clark et al., 2008	Total (42)	Start of pre-SEA	26.0 ± 4.3	179 ± 7	81.3 ± 9.9	12.6 ± 3.0	-	-	-	-	-	-
		Start of pre-SEA	25.0 ± 3.1	179 ± 5	81.0 ± 9.7	12.8 ± 3.0	-	-	-	-	-	-
		Start of pre-SEA	25.0 ± 3.4	177 ± 4	77.9 ± 4.3	11.8 ± 2.3	-	-	-	-	-	-
Svantesson et al., 2008	Total (17)	During competitive SEA	24.1 ± 3.8	183.5 ± 6.4	80.6 ± 7.7	9.7 ± 3.6	-	-	-	-	72.8 ± 7.9	-
Hoppe et al., 2013	Total (11)	Start of pre-SEA	23.8 ± 3.0	179.2 ± 8.9	76.6 ± 8.6	9.6 ± 3.1	-	-	-	-	-	-
Suda et al., 2013	Total (21)	End of pre-SEA	24.7 ± 5.2	177.8 ± 6.2	72.5 ± 6.9	12.3 ± 2.6	9.0 ± 2.5	-	-	-	63.4 ± 5.0	-
Micheli et al., 2014	Total (219)	1st half of SEA	26.1 ± 4.4	181.9 ± 6.3	79.2 ± 6.7	16.1 ± 2.0	12.9 ± 2.4	-	-	-	66.3 ± 4.9	47.9 ± 4.1
Mascherini et al., 2015	Total (59)	Start of pre-SEA	22.47 ± 5.58	181 ± 8	76.48 ± 8.35	-	-	-	-	-	64.59 ± 6.82	47.34 ± 5.01
Semjon et al., 2016	Total (120)	6 consecutive pre-SEA	-	-	-	-	-	-	-	-	-	-

	Central defenders (18)		27.3 ± 6.2	187.9 ± 4.9	84.5 ± 4.6	11.7 ± 2.8	-	-	-	-	-	-
	Lateral defenders (15)		26.7 ± 4.8	180.7 ± 3.6	76.5 ± 4.8	11.2 ± 2.4	-	-	-	-	-	-
	Central midfielders (24)		25.8 ± 5.3	181.2 ± 5.8	75.8 ± 6.3	9.9 ± 2.9	-	-	-	-	-	-
	Wingers (18)		25.3 ± 4.2	177.6 ± 4.4	74.6 ± 5.1	10.3 ± 2.1	-	-	-	-	-	-
	Forwards (34)		24.0 ± 3.6	183.3 ± 7.2	79.4 ± 7.7	10.4 ± 3.1	-	-	-	-	-	-
	Goalkeepers (11)		26.6 ± 6.5	188.6 ± 3.3	87.0 ± 4.6	11.5 ± 3.1	-	-	-	-	-	-
Aras et al., 2017	Total (12)	-	18.33 ± 0.98	178.83 ± 4.57	71.68 ± 4.82	15.76 ± 3.51	-	-	-	-	-	-
Requena et al., 2017	Total (19)	Start of pre-SEA	26.2 ± 2.8	180.3 ± 3.3	76.9 ± 6.3	11.6 ± 3.6	-	-	-	-	-	-
Kafedžić et al., 2018	Total (39)	2 start of pre-SEA	23.5 ± 4.6	182.0 ± 5.6	77.8 ± 5.8	10.3 ± 3.2	-	45.3 ± 2.8	-	-	-	-
Marcos et al., 2018	Total (233)	Start of pre-SEA	25.37 ± 5.06	178.92 ± 6.23	76.81 ± 7.17	12 ± 3	-	-	-	-	-	-
Suarez-Arrones et al., 2018	Total (18)	End of SEA	27.6 ± 3.0	183.9 ± 6.6	78.3 ± 4.5	9.5 ± 2.6	-	-	-	-	-	-
Clemente et al., 2019	Total (23)	Start of pre-SEA	24.7 ± 2.8	179.2 ± 6.3	76.75 ± 5.56	14.04 ± 1.12	-	-	-	85.96 ± 1.12	-	-
Gardasevic et al., 2019	Total (70)	End of SEA	22.84 ± 4.47	182.67 ± 6.16	78.45 ± 7.60	10.00 ± 2.95	-	-	39.89 ± 3.57	-	-	-
Pietraszewska et al., 2019	Total (29)	During of competitive SEA	25.6 ± 5.8	183.0 ± 6.0	76.7 ± 8.0	18.2 ± 3.0	14.0 ± 3.1	59.4 ± 3.1	45.5 ± 4.8	81.8 ± 3.0	62.7 ± 5.9	-
Campa et al., 2020	Total (176)	-	27.4 ± 4.3^{GD}<br>28.0 ± 5.0^{GV}	183.8 ± 0.5^{GD}<br>182.9 ± 0.5^{GV}	79.5 ± 6.1^{GD}<br>78.9 ± 6.5^{GV}	13.3 ± 1.9^{GD}<br>12.9 ± 1.9^{GV}	10.6 ± 1.7^{GD}<br>10.2 ± 1.9^{GV}	-<br>-	-<br>-	-<br>-	68.8 ± 5.4^{GD}<br>68.7 ± 5.7^{GV}	-<br>-
Dağcilar & Öztürk, 2020	Total (191)	During of competitive SEA	24.7 ± 5.5	177.8 ± 6.1	75.2 ± 8.1	9.1 ± 3.3	-	-	-	-	68.2 ± 6.2	47.3 ± 3.0
Gardasevic & Bjelica, 2020	Total (53)	End of SEA	22.75 ± 4.16	180.30 ± 5.56	75.18 ± 7.24	9.83 ± 3.18	-	-	38.31 ± 3.15	-	-	-
Granero-Gil et al., 2020	Total (30)	During of competitive SEA	26.57 ± 5.56	182 ± 5	77.20 ± 2.76	8.44 ± 1.08	-	-	-	-	-	-
Książek et al., 2020	Total (26)	Pre-SEA	27.0 ± 3.7	190 ± 1	78.3 ± 6.9	19.5 ± 3.3	15.6 ± 3.1	58.8 ± 3.4	47.0 ± 4.8	80.4 ± 3.5	64.3 ± 6.4	47.2 ± 4.6
Radzimiński et al., 2020	Total (23)	During of competitive SEA	27.9 ± 4.58	181.7 ± 6.53	78.8 ± 7.35	9.6 ± 2.2	-	-	-	-	-	-
Castro Jiménez et al., 2021	Total (24)	-	21.0 ± 1.9	177.3 ± 4.3	73.21 ± 6.42	-	-	-	-	-	-	-
	Defenders (5)		-	181.7 ± 6.1	75.3 ± 8.3	13.0 ± 3.0	-	-	37.4 ± 3.6	-	-	-

	Midfielders (13)		-	175.0 ± 4.6	69.7 ± 4.3	14.4 ± 3.0	-	-	34.6 ± 3.7	-	-	-
	Forwards (4)		-	174.3 ± 4.0	76.4 ± 5.5	15.2 ± 4.3	-	-	37.0 ± 2.2	-	-	-
	Goalkeepers (2)		-	185.2 ± 5.2	80.9 ± 0.8	12.6 ± 5.7	-	-	40.5 ± 2.7	-	-	-

GD=Development group; GV=Validation group; SEA=Season

Table S3

**Authors and years**	**Sample size (n)**	**Time of the SEA**	**Age (years)**	**Height (cm)**	**Weight (kg)**	**Fat mass (%)**	**Fat mass (kg)**	**Fat-free mass (%)**	**Fat-free mass (kg)**	**Bone mineral density (g/cm³)**	**Bone mineral content (kg)**
Wittich et al., 2001	Total (42)	3 consecutive pre-SEA	23.2 ± 3.5	176.5 ± 4.5	76.2 ± 5.4	12.2 ± 3.1	9.2 ± 2.8	82.9 ± 3.1	63.1 ± 3.9	-	-
Svantesson et al., 2008	Total (17)	Spring	24.1 ± 3.8	183.5 ± 6.4	80.6 ± 7.7	10.9 ± 3.5	-	-	72.4 ± 6.2	-	-
Reinke et al., 2009	Total (10)	End of SEA	25.3 ± 5.1	184.2 ± 5.9	90.1 ± 5.6	11.9 ± 6.2	10.3 ± 5.6	-	74.4 ± 4.2	-	-
Sutton et al., 2009	Total (64)	-	26.2 ± 4.0	182 ± 7	83.2 ± 7.5	10.6 ± 2.1	-	81.3 ± 2.0	-	1.383 ± 0.100	-
Gerosa-Neto et al., 2014	Total (82)	Pre-SEA	23.6 ± 4.2	179.9 ± 8.1	77.0 ± 12.8	14.0 ± 5.2	11.3 ± 5.1	-	61.9 ± 7.5	1.42 ± 0.84	-
Novack et al., 2014	Total (31)	-	21.48 ± 3.38	181.97 ± 8.11	79.05 ± 9.48	13.68 ± 4.22	10.99 ± 4.06	-	68.05 ± 7.38	-	-
Milanese et al., 2015	Total (29)	3 consecutive SEA	27.5 ± 4.38	185.2 ± 5.85	82.0 ± 5.95	11.2 ± 2.26	8.56 ± 1.96	-	64.82 ± 4.85	1.219 ± 0.081	2.90 ± 0.32
Milsom et al., 2015	Total (27)	3 consecutive SEA	24.1 ± 3.9	182.5 ± 7.1	81.4 ± 8.2	10.0 ± 1.6	7.8 ± 1.6	-	66.9 ± 7.1	-	-
Sánchez-Ureña et al., 2016	Total (106)	-	24.53 ± 4.77	176.7 ± 6.5	75.45 ± 7.2	13.34 ± 4.1	-	-	62.1 ± 5.5	1.34 ± 0.08	3.6 ± 0.4
Devlin et al., 2017	Total (18)	End of pre-SEA	27 ± 5	180.4 ± 7.3	75.6 ± 5.6	12.8 ± 1.9	8.71 ± 1.43	-	-	-	2.71 ± 0.24
Devlin et al., 2017b	Total (18)	Start of pre-SEA	25 ± 5	180.4 ± 7.4	68.53 ± 6.61	14.7 ± 3.0	10.07 ± 2.49	-	-	-	2.70 ± 0.24
Lopez-Taylor et al., 2018	Total (131)	-	23.2 (20.5 – 26.8)	177.5 ± 5.8	73.2 ± 8.0	14.0 (11.9–16.4)	-	-	-	-	-
Suarez-Arrones et al., 2018	Total (18)	End of SEA	27.6 ± 3.0	183.9 ± 6.6	78.3 ± 4.5	14.4 ± 1.3	-	-	-	-	-

Khalladi et al., 2019	Total (111)	During competitive SEA	23.7 ± 4.8	176.9 ± 5.9	71.0 ± 9.9	20.6 ± 4.4	-	-	56.4 ± 6.4	-	-
Randell et al., 2019	Total (16)	1st pre-SEA	25 ± 4	180.2 ± 7.1	74.3 ± 6.5	13.5 ± 1.8	10.7 ± 2.0	-	60.9 ± 5.4	-	-
		2nd pre-SEA	26 ± 4	180.2 ± 7.1	74.6 ± 6.0	13.6 ± 1.8	10.3 ± 1.5	-	61.4 ± 5.1	-	-
Suarez-Arrones et al., 2019	Total (10)	Start of pre-SEA	27.3 ± 2.8	183.0 ± 8.0	73.2 ± 5.2	15.5 ± 1.4	11.3 ± 1.4	-	61.9 ± 4.4	-	-
McEwan et al., 2020	Total (20)	Start of pre-SEA	25.1 ± 4.1	177.0 ± 6.9	73.75 ± 5.93	14.4 ± 2.3	10.60 ± 1.88	-	59.58 ± 5.27	-	-
Kammerer López et al., 2021	Total (79)	During competitive SEA	23.0 ± 4.4	178.4 ± 6.8	75.2 ± 7.7	13.71 ± 3.18	10.41 ± 2.71	81.60 ± 0.03	61.99 ± 6.59	-	3.46 ± 0.40

SEA=Season
