# Supplementary figures and images for: Body Composition of Male Professional Soccer Players Using Different Measurement Methods: A Systematic Review and Meta-Analysis
Source: Nutrients. 2023 Feb 25;15(5):1160. doi: 10.3390/nu15051160 (PMC10005265; doi:10.3390/nu15051160)

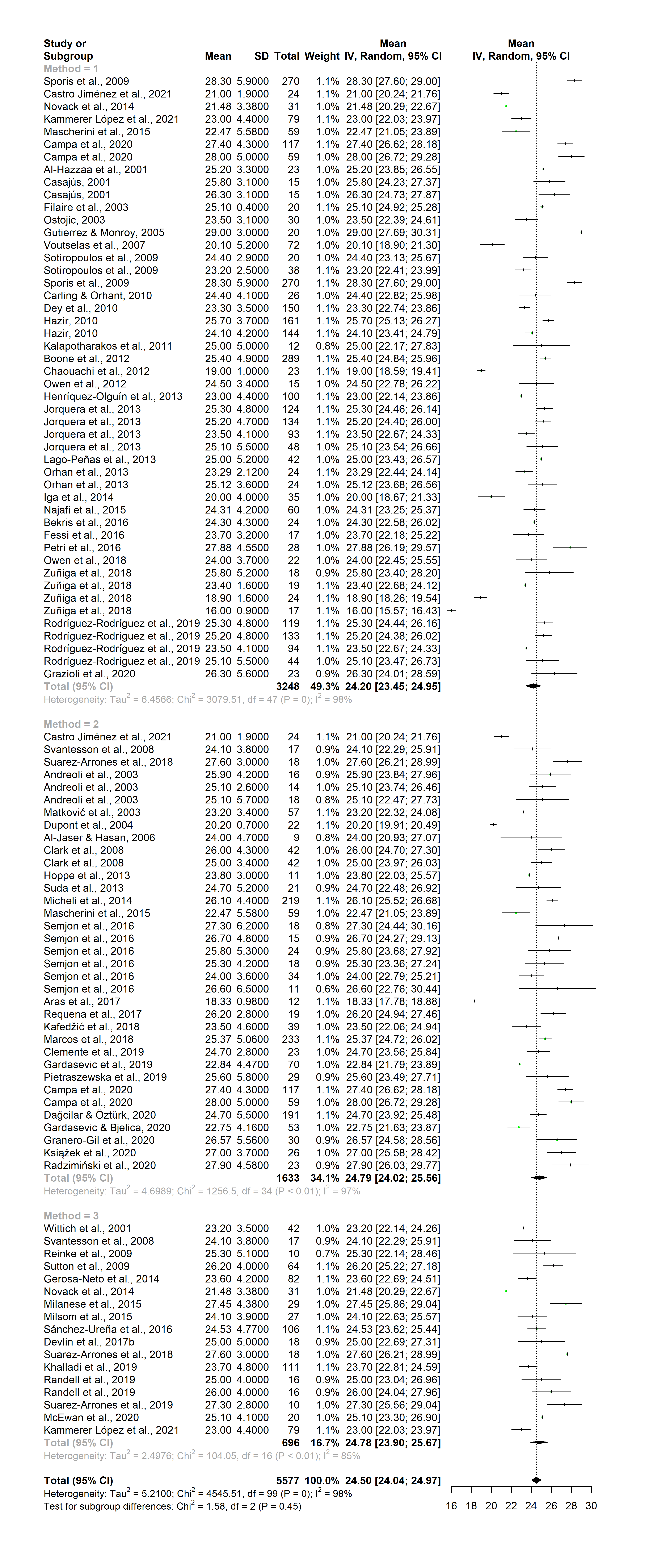

Supplement: Supplementary file 1 [file nutrients-15-01160-s001.zip › Figure S1. Forest plot years old.png]

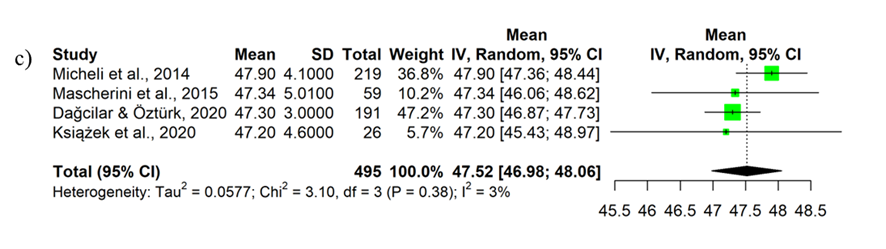

Supplement: Supplementary file 1 [file nutrients-15-01160-s001.zip › Figure S10. Forest plot total body water.png]

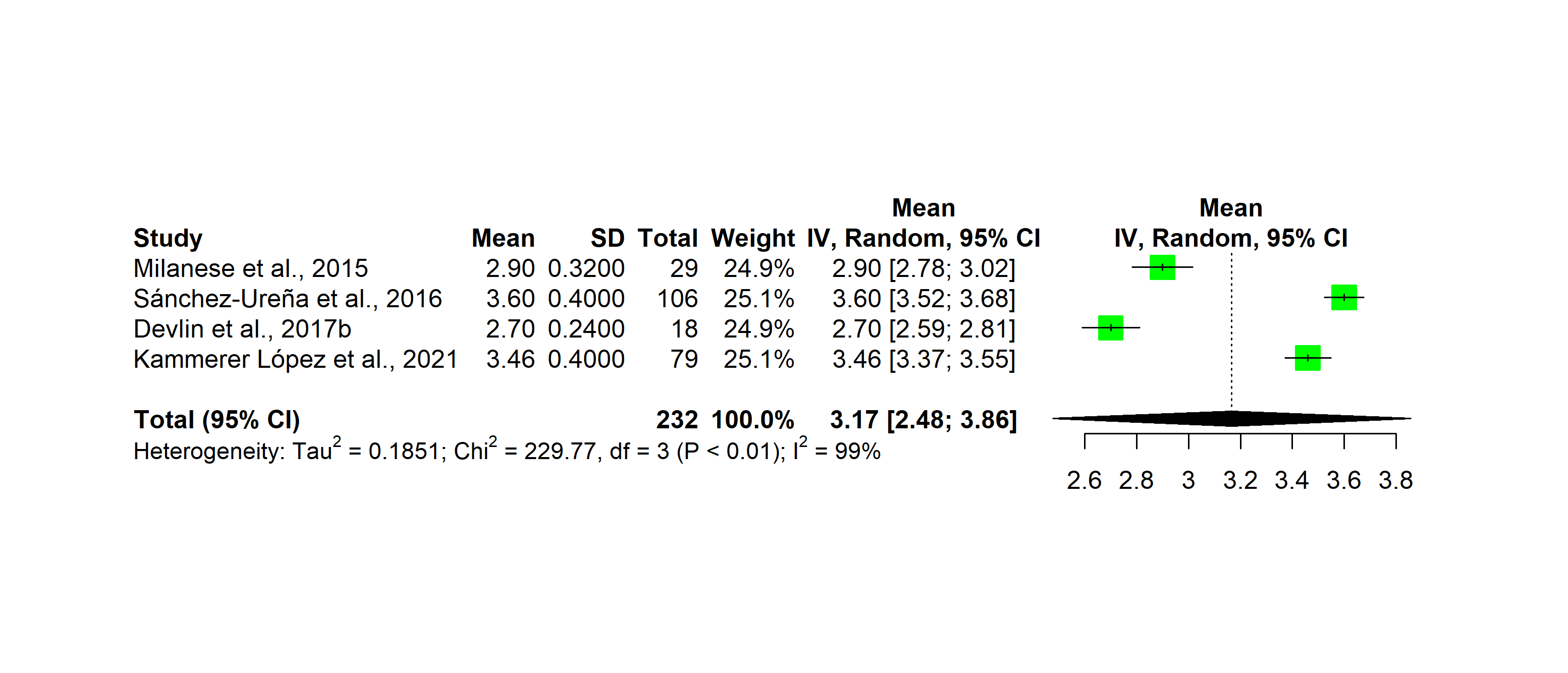

Supplement: Supplementary file 1 [file nutrients-15-01160-s001.zip › Figure S11. Forest plot BMC.png]

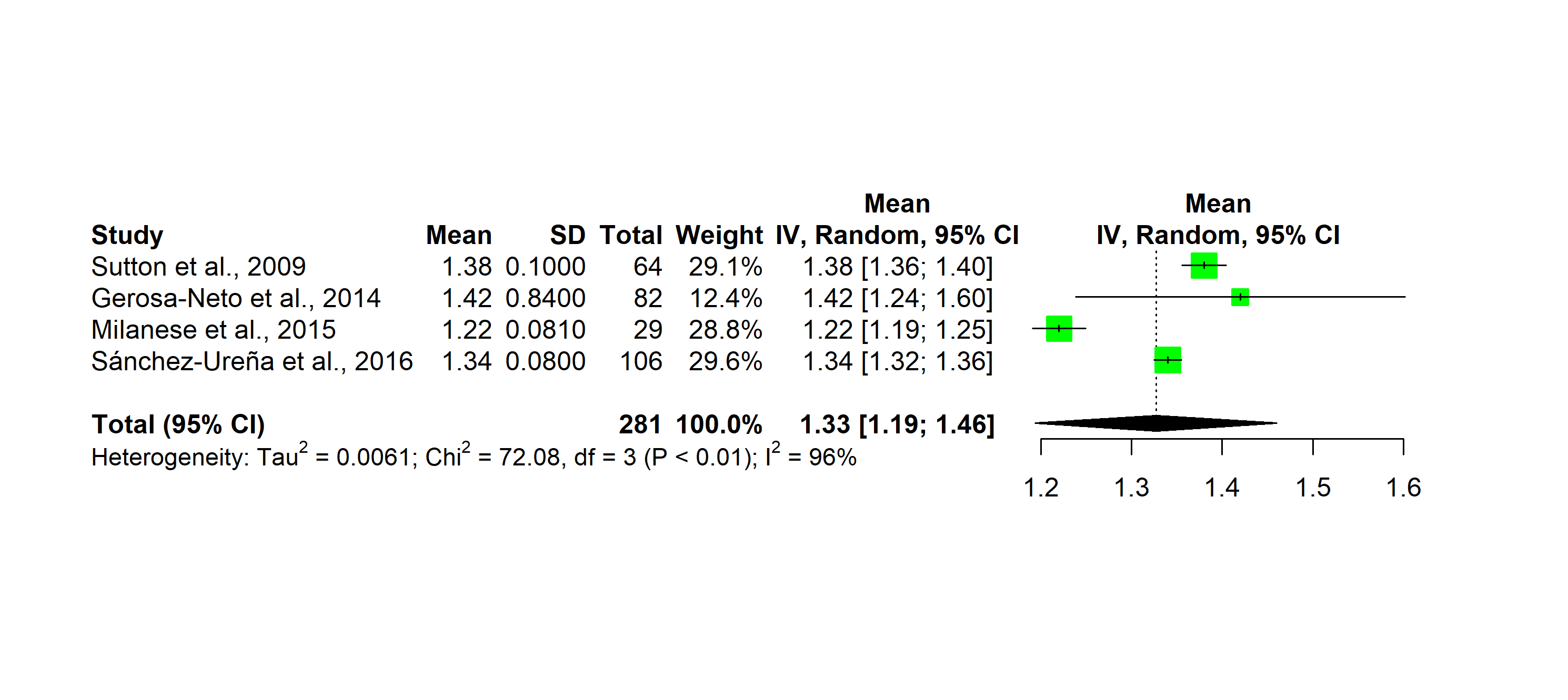

Supplement: Supplementary file 1 [file nutrients-15-01160-s001.zip › Figure S12. Forest plot BMD.png]

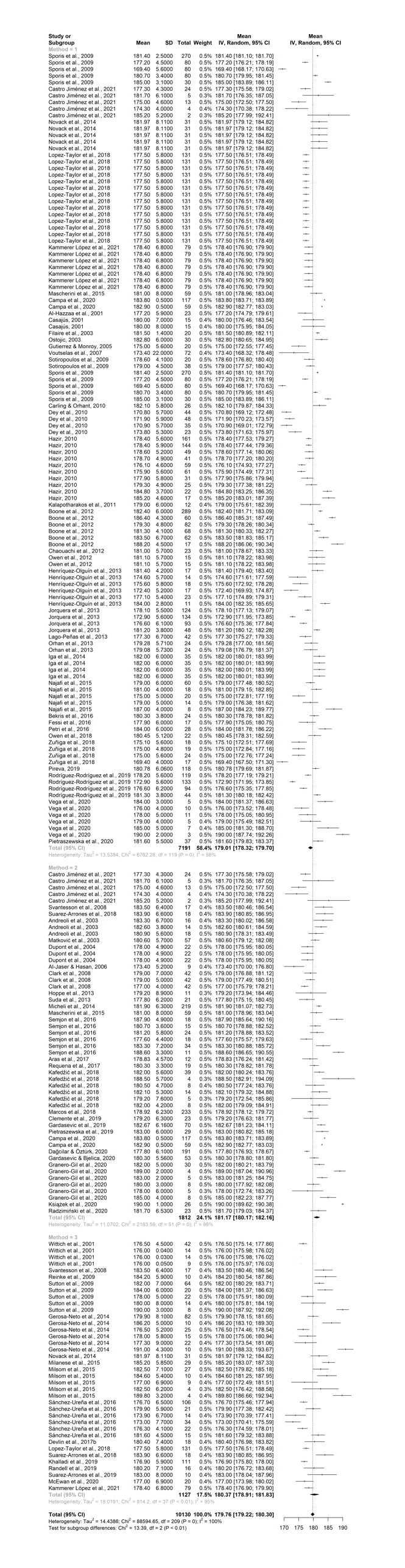

Supplement: Supplementary file 1 [file nutrients-15-01160-s001.zip › Figure S2. Forest plot height.png]

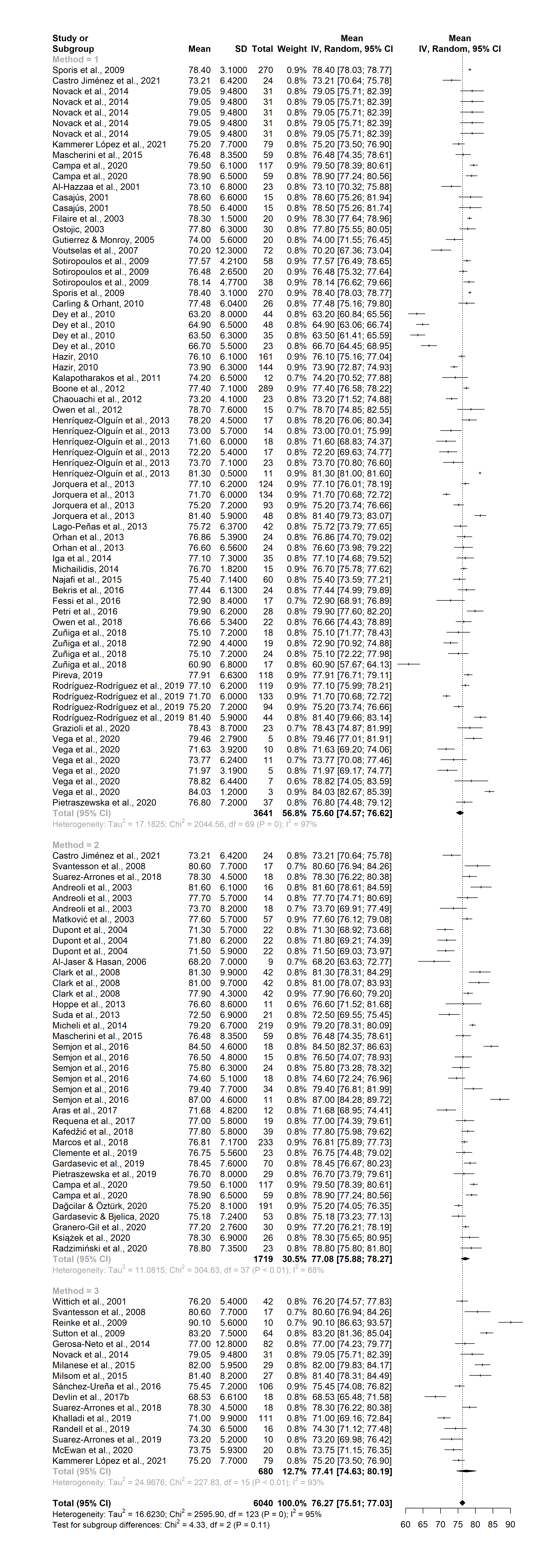

Supplement: Supplementary file 1 [file nutrients-15-01160-s001.zip › Figure S3. Forest plot weight.png]

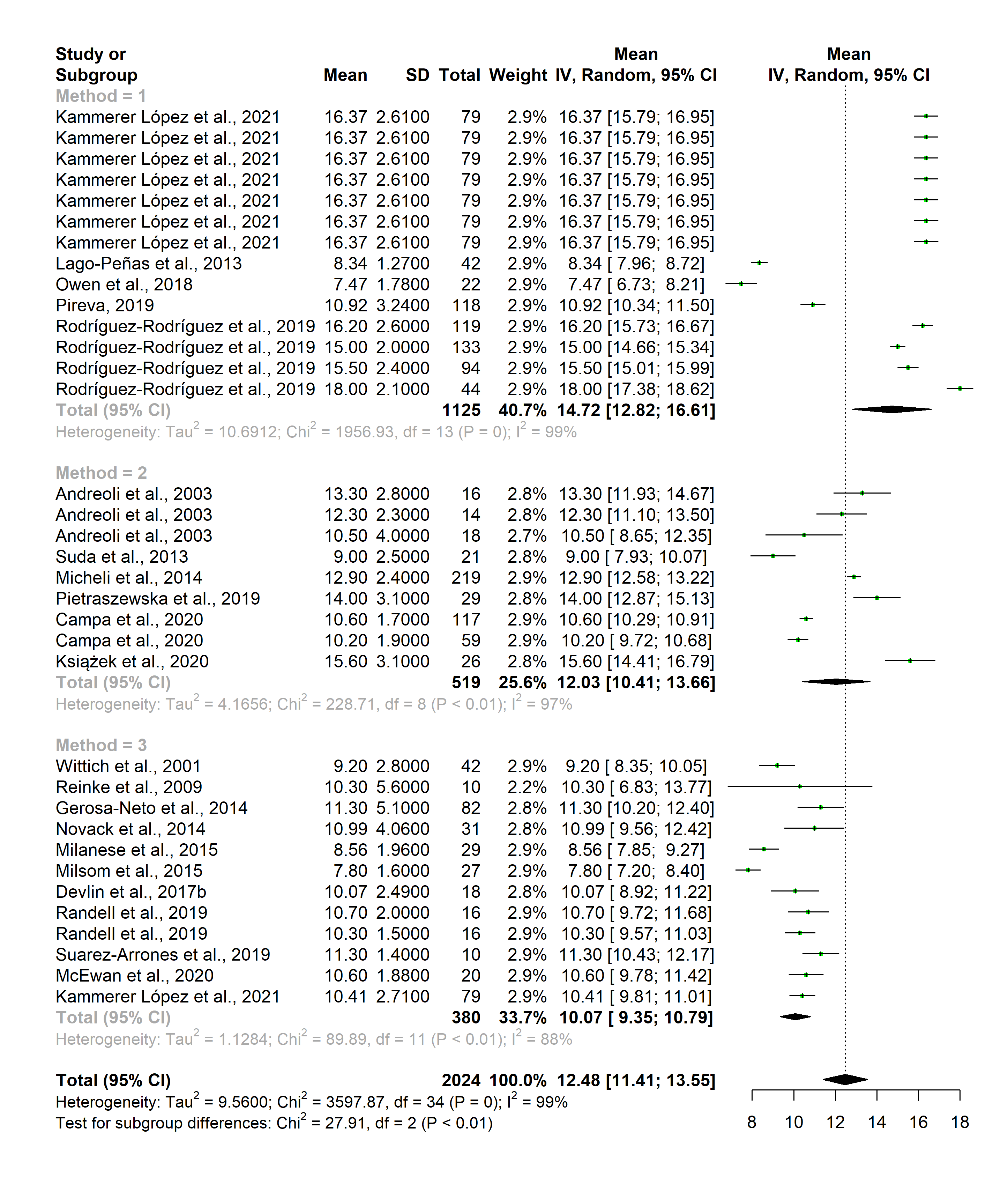

Supplement: Supplementary file 1 [file nutrients-15-01160-s001.zip › Figure S4. Forest plot fat mass kg.png]

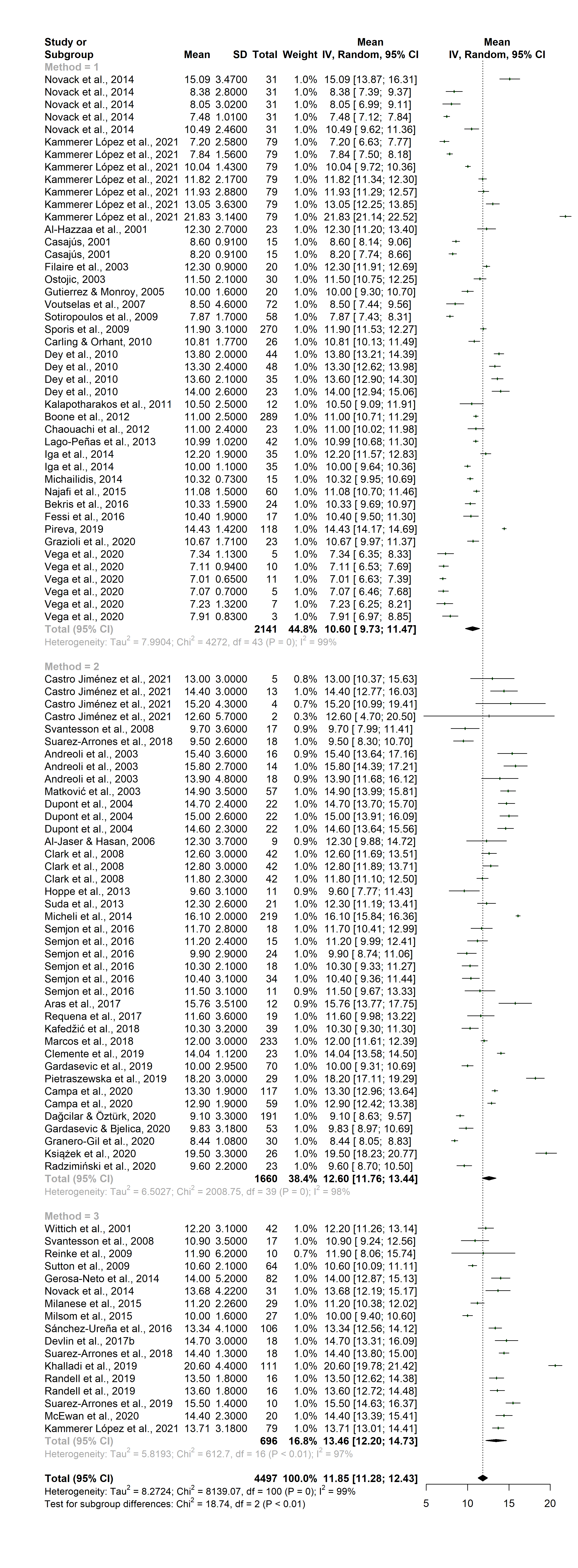

Supplement: Supplementary file 1 [file nutrients-15-01160-s001.zip › Figure S5. Forest plot fat mass %.png]

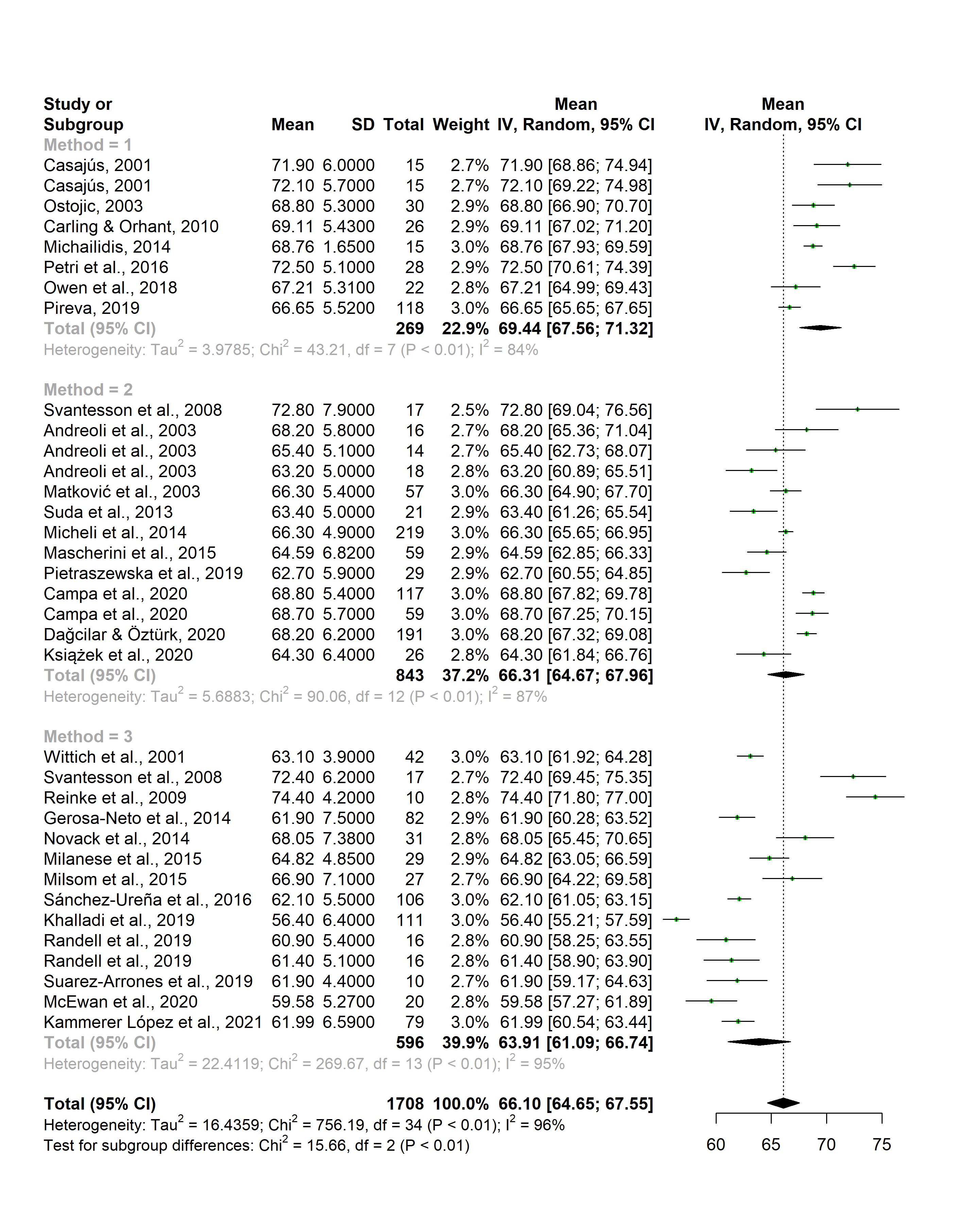

Supplement: Supplementary file 1 [file nutrients-15-01160-s001.zip › Figure S6. Forest plot free mass kg.png]

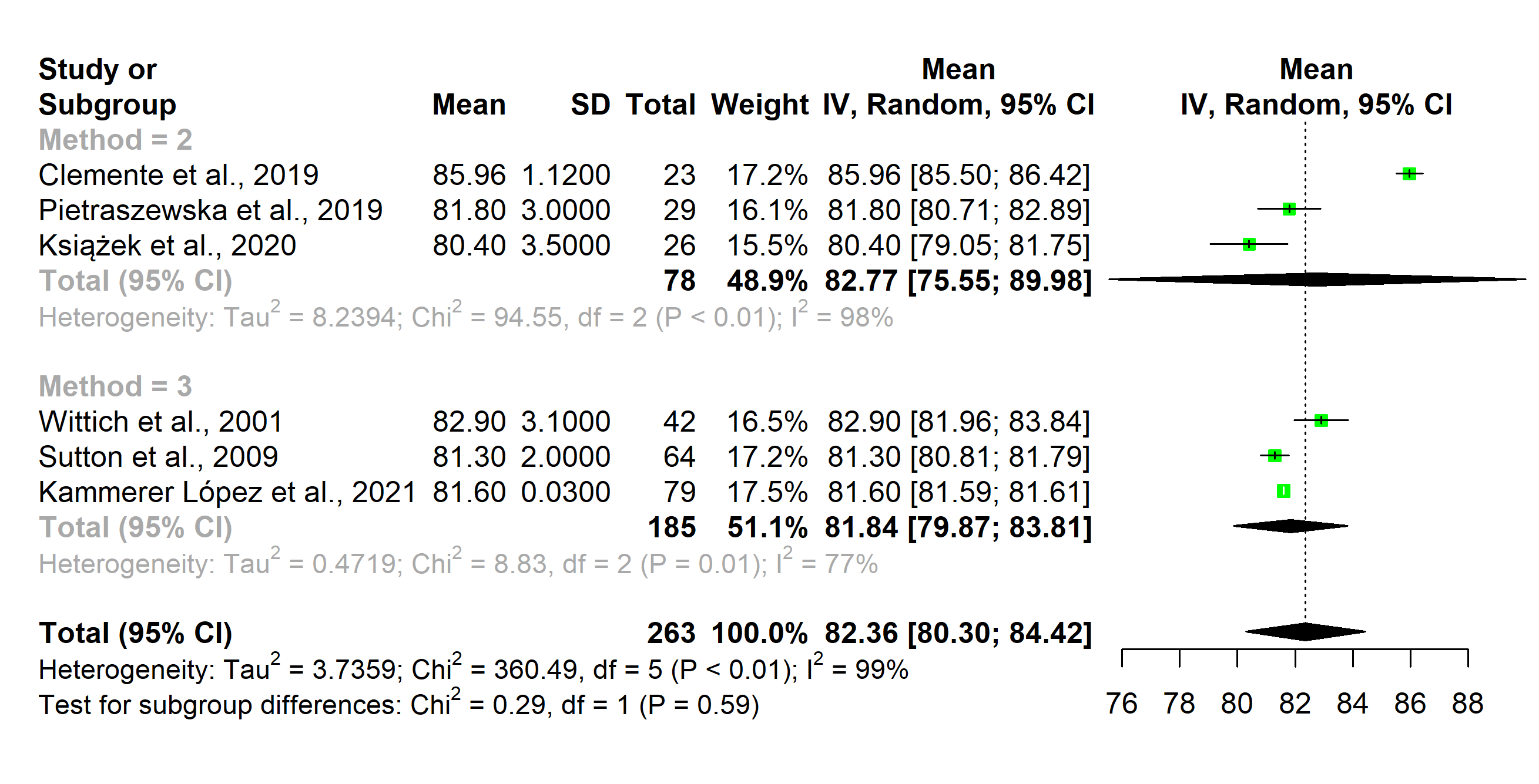

Supplement: Supplementary file 1 [file nutrients-15-01160-s001.zip › Figure S7. Forest plot free mass %.png]

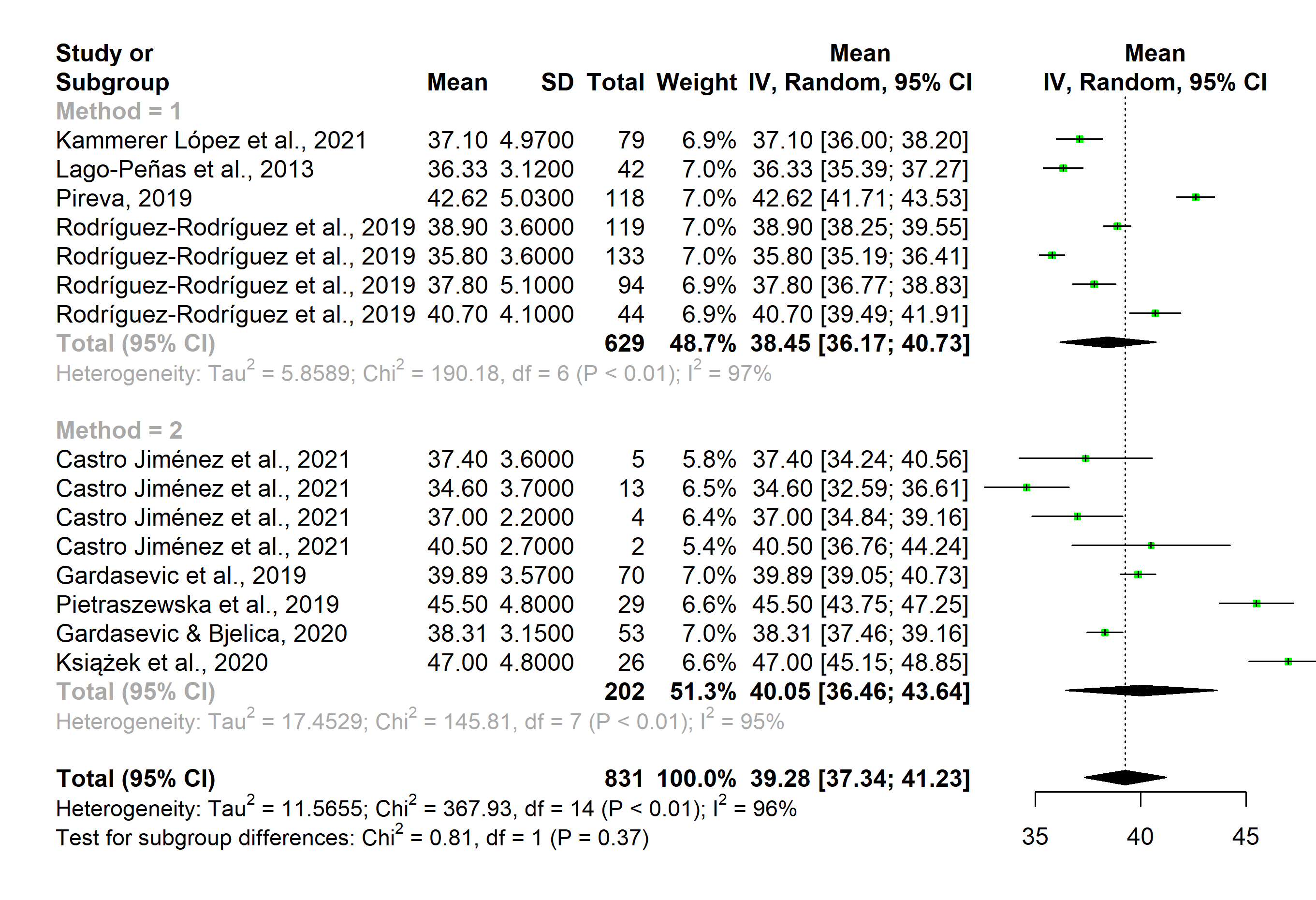

Supplement: Supplementary file 1 [file nutrients-15-01160-s001.zip › Figure S8. Forest plot muscle mass kg.png]

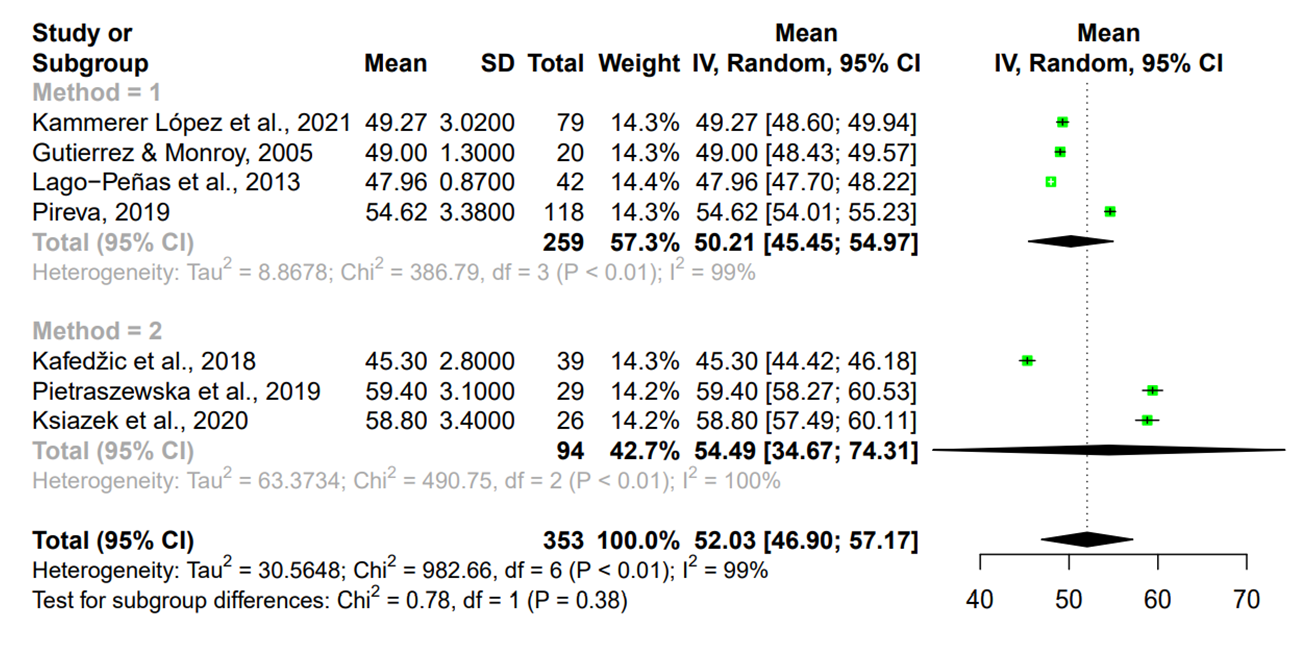

Supplement: Supplementary file 1 [file nutrients-15-01160-s001.zip › Figure S9. Forest plot muscle mass %.png]
